# Gene Expression Signatures Point to a Male Sex-Specific Lung Mesenchymal Cell PDGF Receptor Signaling Defect in Infants Developing Bronchopulmonary Dysplasia

**DOI:** 10.1038/s41598-018-35256-z

**Published:** 2018-11-20

**Authors:** Christina T. Fulton, Tracy X. Cui, Adam M. Goldsmith, Jennifer Bermick, Antonia P. Popova

**Affiliations:** 1 Division of Pediatric Pulmonology, Department of Pediatrics and Communicable Diseases, University of Michigan, Ann Arbor, MI USA; 2 Division of Neonatal-Perinatal Medicine, Department of Pediatrics and Communicable Diseases, University of Michigan, Ann Arbor, MI USA

## Abstract

Male sex is a risk factor for development of bronchopulmonary dysplasia (BPD), a common chronic lung disease following preterm birth. We previously found that tracheal aspirate mesenchymal stromal cells (MSCs) from premature infants developing BPD show reduced expression of PDGFRα, which is required for normal lung development. We hypothesized that MSCs from male infants developing BPD exhibit a pathologic gene expression profile deficient in PDGFR and its downstream effectors, thereby favoring delayed lung development. In a discovery cohort of 6 male and 7 female premature infants, we analyzed the tracheal aspirate MSCs transcriptome. A unique gene signature distinguished MSCs from male infants developing BPD from all other MSCs. Genes involved in lung development, PDGF signaling and extracellular matrix remodeling were differentially expressed. We sought to confirm these findings in a second cohort of 13 male and 12 female premature infants. mRNA expression of PDGFRA, FGF7, WNT2, SPRY1, MMP3 and FOXF2 were significantly lower in MSCs from male infants developing BPD. In female infants developing BPD, tracheal aspirate levels of proinflammatory CCL2 and profibrotic Galectin-1 were higher compared to male infants developing BPD and female not developing BPD. Our findings support a notion for sex-specific differences in the mechanisms of BPD development.

## Introduction

Bronchopulmonary dysplasia (BPD) is the most common pulmonary complication of premature birth and its incidence continues to increase especially among extremely premature infants^1–3^. Survivors of BPD have chronic respiratory symptoms and abnormal lung function with airflow obstruction during childhood and as adults^4–7^. Despite the long-term impact on pulmonary health, strategies to prevent or treat BPD are limited due to incomplete understanding of the mechanisms of BPD development.

Male premature infants have a higher risk of respiratory complications and development of BPD than female infants^8–11^. Male sex is also associated with higher severity of BPD^12,13^. Between 20 and 32 weeks gestation, the lungs of male infants have a lower histologic index of maturity than the lungs of females of the same gestational age^14^, suggesting there are sex-specific differences in late canalicular and saccular stages of lung development. In a murine model of BPD, male mice are more susceptible to hyperoxia-induced hypoalveolarization and disrupted pulmonary angiogenesis^15,16^. The cause for the male sex predilection is not well understood. It is conceivable that there are sex-specific differences in gene expression during normal lung development or in response to early life exposures associated with preterm birth that lead to different mechanisms of BPD development in male and female premature infants.

Signals from the lung mesenchyme are essential during lung development, including distal lung growth and alveolar formation (reviewed in^17^). During late saccular and alveolar stage of lung development, platelet-derived growth factor receptor-α (PDGFR-α)-expressing mesenchymal cells migrate to the tips of secondary alveolar septa and differentiate into alveolar myofibroblasts that are required for alveologenesis^18–20^. The lungs of infants with BPD demonstrate fewer and larger alveoli, as well as poorly formed secondary crests^21^, indicating interference with the normal ingrowth of secondary septa into larger alveolar saccules. In BPD, alveolar septa are thickened with collagen and α-smooth muscle actin -, transforming growth factor (TGF)-β-positive myofibroblasts^22–25^ and have fewer PDGFR-α-expressing cells in the dysmorphic alveolar septa, suggesting abnormal migration and differentiation of mesenchymal progenitor cells within the interstitia of the terminal air spaces^26^.

PDGF signaling and its downstream mediators have been implicated in normal lung development and extracellular matrix remodeling. For example, PDGF signaling induces mesenchymal cell expression of fibroblast growth factors (FGFs), including FGF-7^27^, which is critical for lung alveolar development^28^. FGF-7 in turn upregulates the expression of Sprouty (SPRY)1^29^, a gene involved in mesenchyme-epithelium interaction during lung development^30^. Furthermore, PDGF signaling promotes Wnt2-Wnt7b cooperative signaling mechanism required for mesenchymal cell differentiation during lung development^31^. Loss of Wnt2 during development, leads to lung hypoplasia^31,32^. In BPD, alveolar septa are thickened with collagen, indicating interference with extracellular matrix organization^25^. PDGF signaling in mesenchymal cells upregulates expression of matrix metalloproteinase-3 (MMP3), a proteolytic enzyme involved in collagen and other extracellular matrix proteins remodeling^33^. Therefore, dysregulation of PDGF receptor signaling in mesenchymal cells may impair distal lung growth and repair through different downstream mediators and thus can contribute to BPD pathogenesis.

We have isolated mesenchymal stromal cells (MSCs) from tracheal aspirates of premature infants with RDS^34^. The gene expression profile of these MSCs is consistent with lung-resident progenitors of alveolar myofibroblasts^35,36^. Isolation of MSCs from tracheal aspirates increases the relative risk of developing BPD by over 20-fold, however not all infants with MSCs go on to develop BPD^37^. Up to this point, we have not examined the sex-specific differences in MSC gene expression.

In this study, we hypothesized that neonatal lung MSCs from male and female infants display unique transcriptomes providing clues to the male sex predilection for BPD development. We found that MSCs from male infants developing BPD display a gene signature with significantly lower expression of genes required for distal lung development and thus appear predisposed for impaired alveologenesis.

## Results

### Patient characteristics

We performed an unsupervised exploratory gene expression analysis using MSCs isolated from tracheal aspirates of 13 premature infants with RDS who required mechanical ventilation in the first week of life (Table 1). Six of the 13 infants were male (46%) and 7 out of the 13 infants (4 male and 3 female, 54%) later developed BPD. BPD was defined by supplemental oxygen requirement at 36 weeks corrected gestational age. In this cohort, infants developing BPD had significantly lower gestational age at birth and a tendency for lower birth weight. There were no statistically significant differences between the gestational age of male and female infants irrespective of outcome. Male infants tended to have higher birth weights than female, but the differences did not reach statistical significance. Also, there were no significant differences in the day of life or FiO2 concentration when the tracheal aspirate was collected between male and female infants as well as between infants developing BPD and the ones not developing BPD.

**Table 1:** Characteristics of patients included in the discovery cohort.

Patient	Sex	Race	Gest. age (wks)	Birth weight (g)	BPD Dx	Vent days	Total O_2_ days	Surf. doses	Chorioamnionitis	DOL sample collected	FiO_2_
1	Female	White/Black	32 3/7	1630	No	1	7	1	No	1	0.21
2	Female	White	29 6/7	1425	No	3	4	2	No	1	0.21
3	Male	White	31 3/7	1620	No	3	5	1	Yes	2	0.21
4	Male	White	28 5/7	1395	No	2	26	1	No	1	0.21
5	Female	Black	29	1220	No	5	37	2	No	2	0.21
6	Female	White	26	570	No	27	70	2	No	1	0.25
7	Male	White	27 6/7	1615	Yes	571	571	2	Yes	2	0.23
8	Male	White	27	1265	Yes	42	160	2	No	2	0.21
9	Female	White	28 5/7	1180	Yes	20	240	3	Yes	0	0.30
10	Male	White	28	1160	Yes	28	61	3	No	2	0.28
11	Female	White	24 4/7	735	Yes	42	87	2	No	2	0.21
12	Female	Black	23 5/7	570	Yes	50	96	3	Yes	4	0.30
13	Male	White	25 3/7	875	Yes	45	131	1	No	2	0.21
Mean,SD			28, 2	1174, 380	7/13	65, 153	115, 153				

DOL, day of life.

### Exploratory gene expression profiling analysis of neonatal lung MSCs

**Figure 1:**
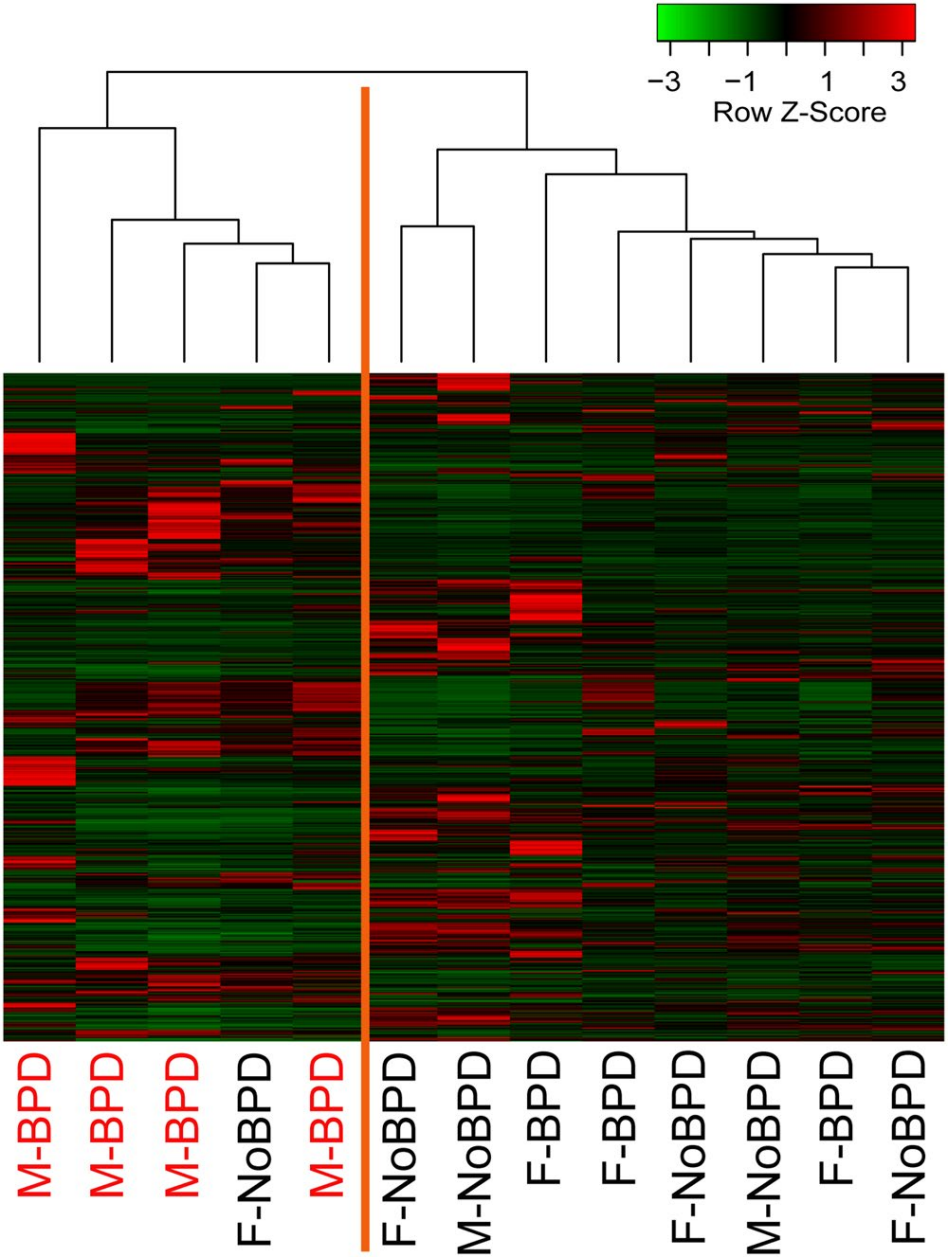
Neonatal lung MSCs expression profiles. Heatmap of unsupervised hierarchical cluster analysis of neonatal lung MSCs expression data based on 870 genes, selected after filtering out genes with low expression levels and low variance. The discovery cohort included 13 infants. Each column represents a sample and each row represents a gene. The Z-score depicts the expression level as a measure of distance, in standard deviations, away from the mean expression of a given gene across all samples. The relative expression value for each gene is depicted by color intensity, with red indicating upregulated and green indicating downregulated genes.

Total RNA from early passage MSCs was isolated and subjected to whole genome transcriptome analysis. Greater than 18,000 unique sequences from the NCBI RefSeq database were tested. Genes with low average expression (less than negative control and noise) and low variance (less than 2 standard deviations outside of the mean gene expression level) were filtered out. The remaining 870 genes were subjected to hierarchical cluster analysis, which revealed two distinct clusters (Fig. 1). All MSCs from male infants developing BPD (M-BPD) clustered together and separately from the MSCs from other male infants who did not go on to develop BPD, as well as from MSCs from all female infants. One MSC isolate from a female infant who did not go on to develop BPD clustered with the M-BPD group.

The clustering of all M-BPD samples together suggested that the transcriptomic profile of these MSCs may hold clues for the higher risk of BPD development and higher BPD severity in male infants. Given the close and non-discriminating clustering of all but one of the rest of the samples, in our subsequent analysis we combined the MSCs from male infants who did not go on to develop BPD, as well as MSCs from all female infants in a second cluster as a Combined Control group. Using an unpaired *t*-test, we compared the expression values of the 870 genes between the M-BPD and Combined Control groups. To better pinpoint the genetic differences we generated a list of 166 genes, selected based on unadjusted *p*-value less than 0.05 and fold change equal or greater than 2. Among these genes we recognized genes involved in distal lung development, such as PDGFRA, FGF7, WNT2, SPRY1 and FOXF2. We also identified genes participating in extracellular matrix formation and remodeling, including MMP3, FBLN1, FBN2, COL4A5, ITGA8 and others (Supplemental Table S1). We analyzed the 166 differentially expressed genes for over-representation of pathways and gene ontology terms using the ConcensusPathDB database (http://cpdb.molgen.mpg.de/)^38,39^. We identified 48 enriched pathway-based sets of genes as defined by the KEGG, Reactome and BioCarta databases (Supplemental Table S2). Among the over-represented pathways relevant to BPD pathogenesis, were pathways related to extracellular matrix organization (12 genes, p-value = 1e-05, q-value = 0.00149), elastic fiber formation (5 genes, p-value = 1.25e-05, q-value = 0.00149), degradation of extracellular matrix (5 genes, p-value = 0.00248, q-value = 0.0428) and signaling by PDGF (8 genes, p-value = 0.00849, q-value = 0.0493) (Table 2). Other over-represented pathways included Wnt signaling pathway, five PI3K/AKT-related pathways, and three EGFR-related signaling pathways (p-values from 0.00252 to 0.00934). We also found 143 enriched gene ontology-based sets of genes (Supplemental Table S3). The over-represented sets included gene ontology terms for biological processes related to BPD development and included: lung morphogenesis (6 genes, p-value = 3.19e-06, q-value = 7.07e-05), respiratory system development (9 genes, p-value = 3.16e-05, q-value = 0.000341), regulation of cell proliferation (34 genes, p-value 1.12e-08, q-value = 2.68e-06), regulation of cell motility (22 genes, p-value = 3.38e-08, q-value = 2.68e-06), vasculature development (20 genes, p-value = 4.61e-08, q-value = 2.68e-06), mesenchyme development (13 genes, p-value = 3.98e-08, q-value = 2.68e-06) (Table 3).

**Table 2:** Over-represented pathways relevant to BPD pathogenesis.

Pathway name	p-value	q-value	Genes
Extracellular matrix organization	1.00E-05	0.00149	ITGA8, ADAMTS5, BMP4, ADAMTS1, COL4A5, FBN2, ADAMTS8, NID1, LTBP4, COL21A1, FBLN1, MMP3
Elastic fiber formation	1.25E-05	0.001485	ITGA8; BMP4; FBLN1; LTBP4; FBN2
Degradation of the extracellular matrix	0.00248	0.042772	ADAMTS5; NID1; ADAMTS1; ADAMTS8; MMP3
Signaling by PDGF	0.008485	0.049294	PDGFRA; TEK; PDGFD; COL4A5; GFRA1; FGF9; FGF7; KITLG

**Table 3:** Over-represented gene ontology terms for biological processes related to BPD development.

GO:term ID	GOterm name	p-value	q-value	Genes
GO:0060425	lung morphogenesis	3.19E-06	7.07E-05	BMP4, WNT2, SPRY1, RDH10, FGF7, TCF21
GO:0060541	respiratory system development	3.16E-05	0.000341	BMP4, WNT2, SPRY1, RDH10, FGF9, FGF7, LEF1, ASS1, TCF21
GO:0042127	regulation of cell proliferation	1.12E-08	2.68E-06	PTH1R, PDGFRA, WFDC1, PTGS1, DACH1, SPRY1, BCHE, TPM1, IL1A, FBLN1, LEF1, PTGES, PODN, TEK, ADAMTS1, TWIST1, TFAP2C, ADAMTS8, PTGER2, CDH2, PDE5A, TGFBR3, PDGFD, DLL1, SERPINF1, FGF9, FGF7, KITLG, CXCL12, BMP4, ETV5, SEMA5A, CCND1, WNT2
GO:2000145	regulation of cell motility	3.38E-08	2.68E-06	RECK, PDGFRA, DACH1, FBLN1, SEMA4D, KITLG, PODN, TEK, TWIST1, TPM1, PDGFD, CYGB, SERPINF1, FGF7, LEF1, CXCL12, PLXNC1, SEMA3C, BMP4, PLD1, SEMA5A, RARRES2
GO:0001944	vasculature development	3.81E-08	2.68E-06	RECK, PDGFRA, BMP4, PDGFD, SEMA5A, PRICKLE1, TWIST1, WNT2, TSPAN12, SEMA3C, ARHGAP22, TEK, SERPINF1, FGF9, IL1A, LEF1, CDH2, TNFRSF12A, TCF21, DLL1
GO:0060485	mesenchyme development	3.98E-08	2.68E-06	TGFBR3, SEMA3C, BMP4, FGF9, SEMA5A, TWIST1, WNT2, RDH10, FOXF2, LEF1, KITLG, SEMA4D, TCF21

### Lung MSCs from male infants developing BPD express lower levels of genes involved in distal lung development

**Figure 2:**
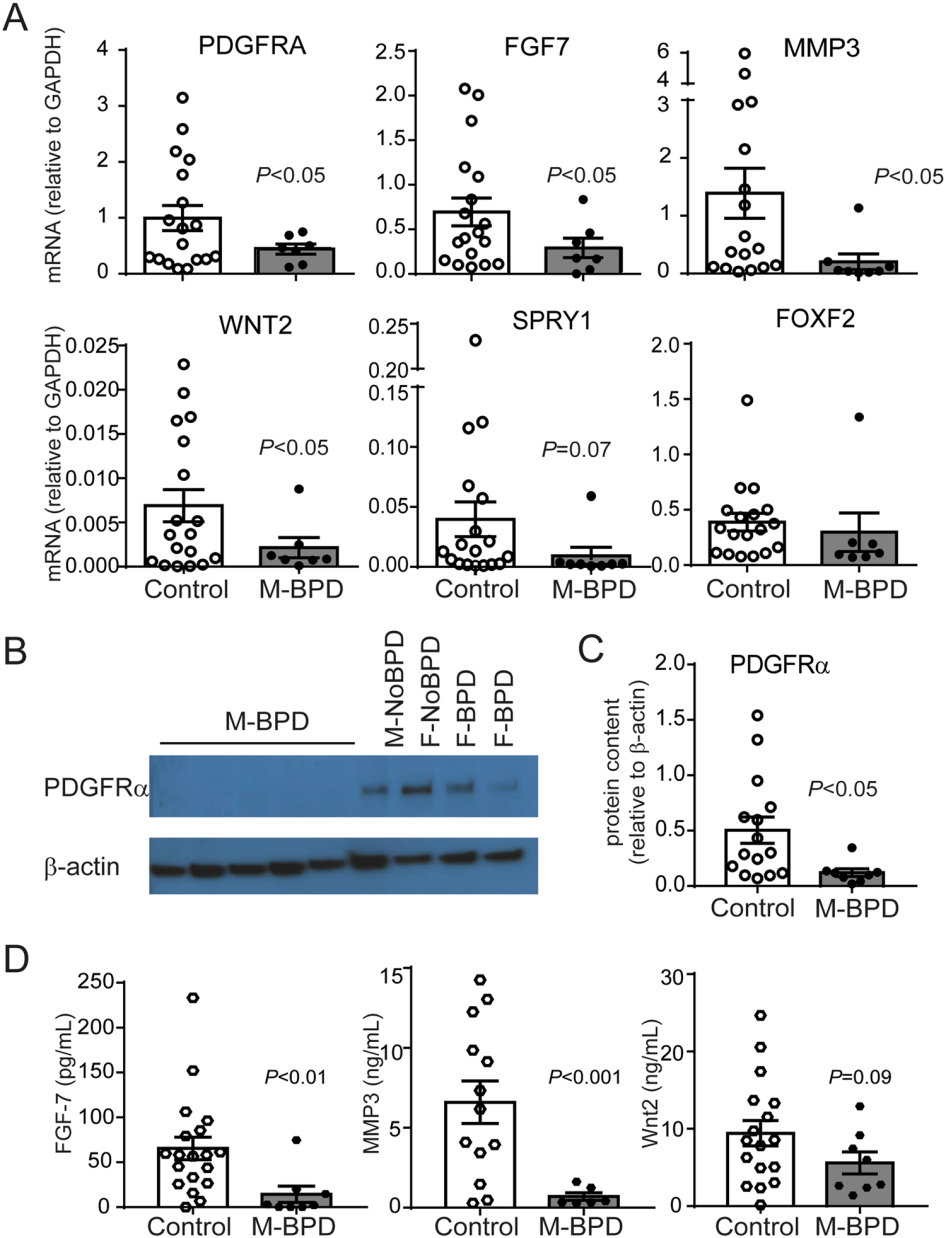
Validation of the differential gene expression between MSCs from male infants developing BPD and combined controls. mRNA expression was assessed by quantitative PCR and protein expression was assessed by immunoblotting and ELISA. (**A**) Compared to the control group MSCs from male infants developing BPD showed significantly lower expression of PDGFRA, FGF7, WNT2 and MMP3 and a trend for lower SPRY1 and FOXF2 mRNA expression. (**B**) Representative immunoblot confirms decreased protein expression of PDGFRα. MSCs from 5 male infants developing BPD and 4 control infants (one male and one female infants who were not developing BPD and two female infants developing BPD) are shown. Full-length blots are available in Supplemental Fig. S1. (**C**) PDGFRα densitometry analysis group mean data for 9 male infants developing BPD and 18 controls. (**D**) MSCs from male infants developing BPD secrete significantly lower concentrations of FGF-7, MMP3 and Wnt2. Data are means ± SE. Statistical significance was determined by unpaired *t*-test.

To validate the expression of selected differentially expressed genes identified by our exploratory transcriptomic analysis, we analyzed MSCs from a second cohort of 25 premature infants with RDS (Table 4). Total thirteen of 25 infants were male (52%) and 15 of the 25 (7 male and 8 female infants) developed BPD. In this cohort, there were no significant differences in day of life when the tracheal aspirate was collected between male and female infants, as well as between infants developing BPD and the ones not developing BPD. While the FiO2 concentration at the time of sample collection was higher in infants developing BPD compared to the infants not developing BPD, there was no significant difference between male and female infants developing BPD. The genes selected for this validation analysis were chosen for known relationship to respiratory system development, PDGFR-related signaling and extracellular matrix organization, and included PDGFRA, FGF7, WNT2, SPRY1, MMP3, FOXF2. Based on our observation from the exploratory transcriptomic analysis, as described above, we compared the expression of these genes between the M-BPD and Combined Controls groups (Fig. 2). We found significantly lower mRNA expression of PDGFRA, WNT2, FGF7 and MMP3 in M-BPD isolates compared to the Combined Controls. SPRY1 and FOXF2 mRNA expression was also decreased in the M-BPD isolates, but the differences did not reach statistical significance. In addition, proteins levels of PDGFRα, by immunoblot, and FGF7 and MMP3, by ELISA, were significantly lower in M-BPD isolates compared to the Combined Controls. Wnt2 protein levels also tended to be lower in M-BPD isolates. Together these results confirmed that M-BPD isolates expressed significantly lower levels of genes involved in lung development and extracellular matrix organization.

**Table 4:** Characteristics of patients included in the second cohort for validation analysis.

Patient	Sex	Race	Gest. age (wks)	Birth weight (g)	BPD Dx	Vent days	Total O_2_ days	Surf. Doses	Chorioamnionitis	DOL sample collected	FiO_2_
1	Male	White	26	910	Yes	44	262	2	No	3	0.30
2	Male	White	24 5/7	950	Yes	32	259	2	No	2	0.21
3	Male	White	28	800	Yes (deceased)	69	69	3	No	2	0.23
4	Male	White	23 5/7	680	Yes (deceased)	47	386	2	No	3	0.21
5	Male	White	26 1/7	815	Yes	79	365	4	No	2	0.28
6	Male	White	25 6/7	910	Yes	49	185	4	No	2	0.30
7	Male	White	24 5/7	745	Yes	50	308	2	No	4	0.21
8	Male	Black	28 3/7	1065	No	26	53	2	No	3	0.28
9	Male	White	25 3/7	900	No	11	38	1	No	2	0.21
10	Male	White	31 6/7	1535	No	2	9	1	No	1	0.21
11	Male	White	29 1/7	1065	No	6	11	1	No	4	0.21
12	Male	White	30 2/7	1640	No	3	6	2	No	3	0.21
13	Male	White	28	1560	No	7	48	2	No	1	0.21
14	Female	White	24 5/7	635	Yes (deceased)	109	109	2	No	2	0.21
15	Female	White	28 5/7	1040	Yes	17	52	2	No	3	0.30
16	Female	Black	26	865	Yes	22	86	1	No	7	0.21
17	Female	Black	25 2/7	855	Yes	unknown	>300	1	Yes	7	0.28
18	Female	Black	27 4/7	835	Yes	32	189	3	No	1	0.21
19	Female	White	27 2/7	935	Yes	16	63	2	Yes	4	0.40
20	Female	White	25 5/7	1035	Yes	42	136	2	Yes	5	0.25
21	Female	White	28 5/7	1210	Yes	22	177	3	Yes	1	0.36
22	Female	Black	28 3/7	1160	No	1	27	1	No	1	0.21
23	Female	Black	29 2/7	1080	No	2	24	1	No	1	0.21
24	Female	White	28 3/7	1080	No	5	7	2	No	2	0.23
25	Female	White	26 5/7	950	No	31	55	2	No	2	0.21

DOL, day of life.

**Figure 3:**
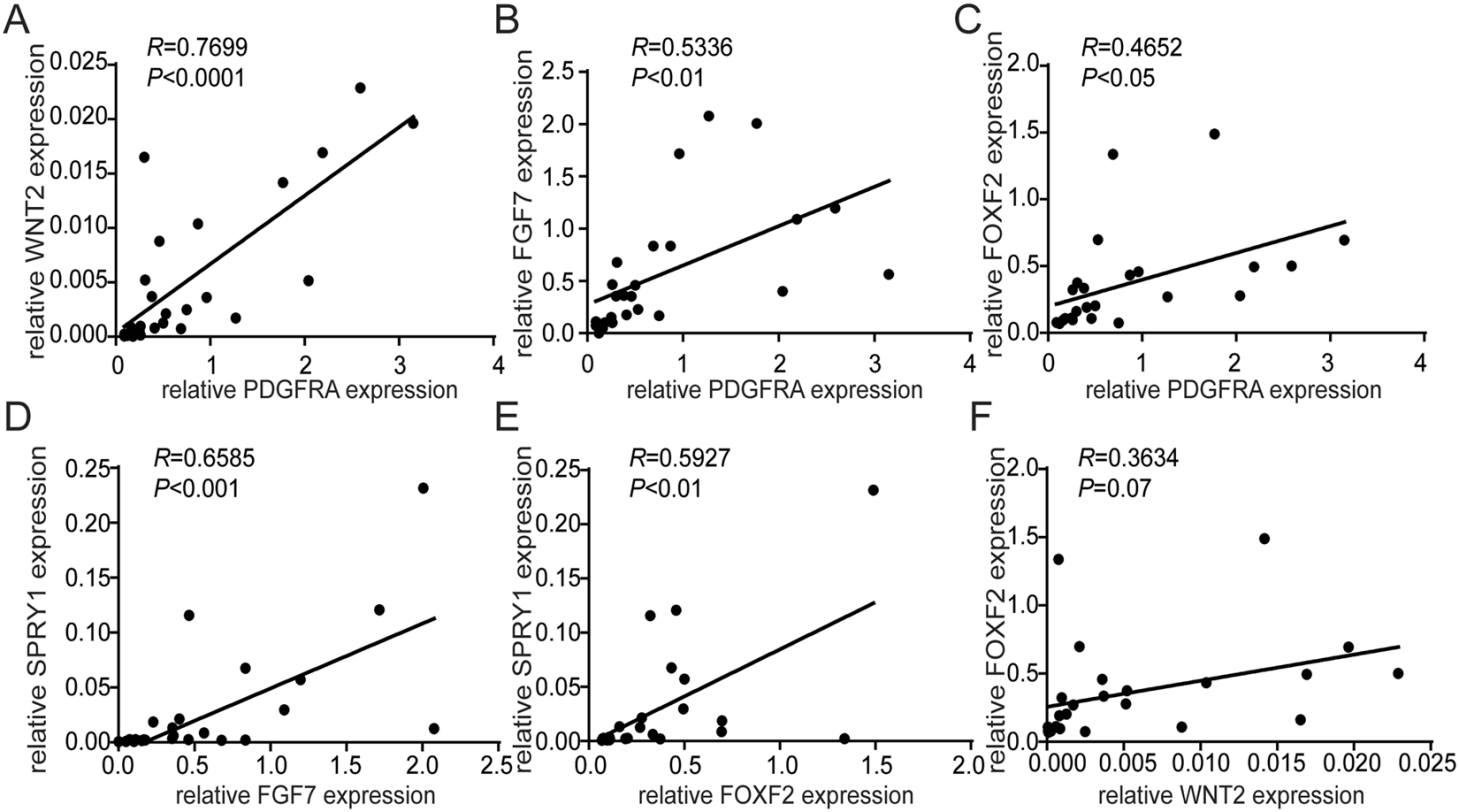
Correlation analysis of lung development gene expression levels. The relationship between the mRNA expression levels of PDGFRA, FGF7, WNT2, SPRY1 and FOXF2 was analyzed by Pearson correlation analysis.

Next, we investigated the relationship between the expression of these genes by correlation analysis. We observed that PDGFRA mRNA expression levels positively correlated with WNT2, FGF7 and FOXF2 mRNA expression levels (Fig. 3A–C). In addition, FGF7 and FOXF2 mRNA expression levels positively correlated with SPRY1 expression (Fig. 3D,E) and WNT2 expression positively correlated with FOXF2 expression (Fig. 3F). Together these results indicate that these genes share a common regulatory pathway. This pathway is disrupted in MSCs from male preterm infants developing BPD.

### Tracheal aspirates from female premature infants developing BPD contain higher levels of CCL2 and the matricellular protein Galectin-1

**Figure 4:**
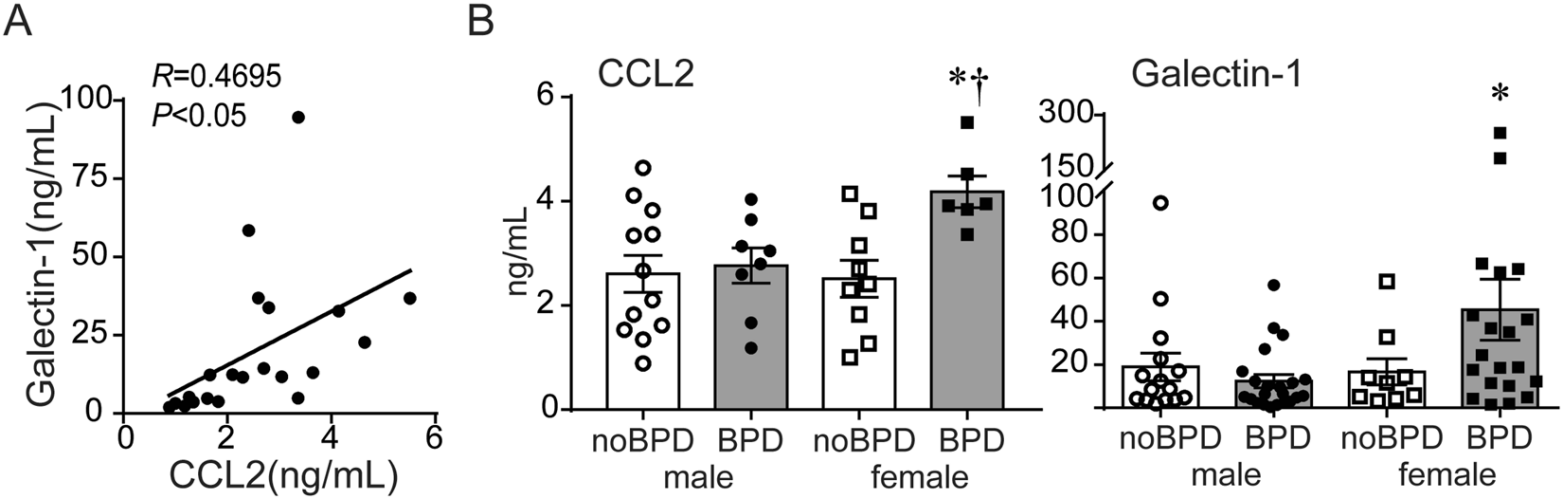
CCL2 and Gal-1 protein levels in tracheal aspirates of premature infants with RDS. (**A**) The relationship between tracheal aspirate CCL2 and Gal-1 protein levels was analyzed by Pearson correlation analysis. (**B**) In tracheal aspirates from premature infants requiring mechanical ventilation for RDS, CCL2 and Gal-1 protein concentrations were significantly higher in female infants developing BPD compared to all other infants. Data are means ± SE. *p < 0.05 versus male infants developing BPD, ^†^p < 0.05 versus female infants not developing BPD (one-way ANOVA).

Unlike MSCs from male infants developing BPD, the transcriptomic profile of MSCs from female infants developing BPD was not distinctly different from the MSCs from both female and male infants, who did not go on to develop BPD. This suggested that other cell populations or soluble factors were involved in the development of BPD in female premature infants with RDS. Prior studies have linked higher tracheal aspirate levels of the proinflammatory chemokine (C-C motif) ligand 2 (CCL2) in premature infants with RDS and development of BPD^40^. Chorioamnionitis and sepsis are risk factors for BPD development^41,42^ and amniotic fluid levels of CCL2 are higher in premature infants with intra-amniotic infection compared to the ones without infection^43^. In animal studies, CCL2 is required for hyperoxia-induced hypoalveolarization^44^. CCL2 can stimulate collagen synthesis in fibrocytes recruited to the alveolar space during fibrotic lung injury^45^. Galectin-1 (Gal-1), a matricellular protein capable of modulating CCL2 production^46^ and promoting fibrosis^47^, is found in increased levels in the chorioamniotic membranes of mothers with pre-term pre-labor rupture of membranes with chorioamnionitis^48^. Gal-1 is enriched in septal tips during alveolar stage of lung development^49^, however, it can also promote fibrosis through activation of TGF-β1 signaling^50^. We previously found that tracheal aspirates from premature infants undergoing mechanical ventilation for RDS contain nanogram quantities of Gal-1^51^, but we have not examined if these correlate with tracheal aspirate CCL2 levels and if there are sex-specific differences in tracheal aspirate Gal-1 levels. We measured CCL2 and Gal-1 protein levels in tracheal aspirates from male and female premature infants with RDS. CCL2 levels positively correlated with Gal-1 levels (Fig. 4A). In addition, levels of CCL2 and Gal-1 were significantly higher in tracheal aspirates from female infants developing BPD compared to female infants who were not developing BPD and male infants developing BPD (Fig. 4B). Next, we compared the incidence of clinically diagnosed chorioamnionitis in the male and female infants developing BPD in our cohort. We found that while only one out of 12 (8%) male infants developing BPD had a history of chorioamnionitis, 6 out of 11 (55%) female infants developing BPD had a history of chorioamnionitis. Thus, in our cohort, the odds ratio of having history of chorioamnionitis in the female infants developing BPD was significantly higher than in male infants developing BPD (p < 0.05, Fisher’s exact test; OR 13.2, 95% CI 1.485–162.2). Together, these results suggest that in female infants with RDS, especially those with history of chorioamnionitis, BPD development may be triggered by proinflammatory and pro-fibrotic responses. The specific roles of CCL2 and Galectin-1 in impaired alveolar development and BPD pathogenesis remain to be elucidated.

## Discussion

In this manuscript we examined the expression profile of MSCs isolated from tracheal aspirates of male and female premature infants, who were or were not developing BPD. Using unsupervised exploratory gene expression analysis of neonatal lung MSCs, we identified a distinct transcriptomic signature distinguishing MSCs from male infants developing BPD from MSCs from all other infants. The differentially expressed genes included genes involved in lung and respiratory system development, mesenchyme and vasculature development, regulation of cell proliferation and motility, PDGF signaling and extracellular matrix remodeling. For selected genes, we validated the microarray results in MSCs from a second larger cohort of premature infants with similar clinical profile. MSCs from male infants developing BPD expressed significantly lower mRNA and protein levels of PDGFRA, FGF7, WNT2 and MMP3 compared to the Combined Control group. Also, MSCs from male infants developing BPD tended to have lower SPRY1 and FOXF2 mRNA levels. In addition, we demonstrated a significant correlation between PDGFRA mRNA expression and WNT2, FGF7 and FOXF2 expression, FGF7, FOXF2 and SPRY1 mRNA expression, and WNT2 and FOXF2 mRNA expression. Together these results indicated that in male premature infants with RDS reduced expression of mesenchymal cell genes essential for distal lung growth is associated with BPD development. To further understand the mechanisms leading to BPD development in female premature infants, we investigated the possibility of an inflammatory mechanism and measured tracheal aspirate CCL2 and Gal-1 levels. Tracheal aspirate CCL2 and Gal-1 levels were higher in female premature infants developing BPD compared to male infants developing BPD and all infants who were not developing BPD. In addition, among the infants developing BPD, a higher proportion of female infants had a history of chorioamnionitis. These results implicate CCL2-driven proinflammatory responses in BPD development in female premature infants.

We previously reported that neonatal lung MSCs, isolated from tracheal aspirates of premature infants in the first week of life, express high levels of contractile and extracellular matrix protein genes and after prolonged culture in serum free conditions differentiate into myofibroblasts, a pattern characteristic of myofibroblast progenitor cells^36^. In addition, we pointed out differences in MSC gene expression related to development of BPD, including higher phospho-glycogen synthase kinase-3β, β-catenin, and α-actin content^52^ and lower levels of PDGFR-α^26^ in MSCs from infants developing BPD. In this study we provide evidence for sex-specific differences in MSC expression profile that may explain future disease development. Using MSCs from two different cohorts of patients with similar clinical characteristics, we found a unique gene signature in MSCs from male premature infants developing BPD with significantly lower expression of genes essential for alveolar lung growth, including PDGFRA, FGF7, WNT2, MMP3 and SPRY1. These data suggest that, in male premature infants, MSCs express significantly lower levels of genes required for lung alveolar growth, therefore contributing to the development of BPD.

Among the genes with significantly lower expression in MSCs from male infants developing BPD were PDGFRα, FGF7, WNT2, SPRY1, MMP3 and FOXF2. The role of mesenchymal progenitor cells as alveolar myofibroblast precursors and their PDGF-receptor signaling in normal distal lung development and secondary crest formation has been highlighted by a number of studies. PDGFRα-expressing myofibroblasts spread to the tips of the secondary crests during saccular and alveolar stage of lung development, and are required for alveologenesis^18–20,53–57^. Experimental animal studies have demonstrated that prenatal disruption of PDGFRα signaling in lung mesenchymal cells leads to significantly smaller lung size^58^. PDGFRα-induced FGF7 is a potent proliferation stimulus for type II alveolar epithelial cells^59,60^ and is also required for alveolar growth^28^. Lower FGF-7 concentrations in tracheal aspirates of premature infants with RDS in the first 5 days of life are associated with the development of BPD^61^. In mice, FGF-7 prevents lung epithelial injury, induced by oxidative stress^62,63^ and mechanical ventilation^64^, but does not attenuate hyperoxia-induced hypoalveolarization^63^. Also, FGF-7 enhances compensatory lung alveolar growth after pneumonectomy^65^. PDGF signaling is required for Wnt2-Wnt7b cooperative activity, and dependent lung mesenchymal cell differentiation^31,32^ and airway smooth muscle development^66^. Loss of Wnt2 signaling leads to a reduction in PDGFRα expression in the mesenchyme surrounding the airways in the developing lung^66^. FGF-7 also upregulates the expression of SPRY1^29^, a gene involved in mesenchyme-epithelium interaction during lung development^30^. Sprouty proteins inhibit receptor tyrosine kinases, including PDGF signaling^67,68^ thereby suppressing cell growth and migration. Therefore, lung mesenchymal cells not only contribute to normal distal lung development, but through aberrant PDGFR and Wnt2 signaling may contribute to impaired alveolar growth, a primary feature of BPD.

PDGF signaling in mesenchymal cells upregulates expression of MMP3, a proteolytic enzyme involved in collagen and other extracellular matrix proteins remodeling^33^. In a previous report, premature infants with RDS, who develop BPD have higher MMP-3 levels in bronchoalveolar lavage fluid compared to infants who do not develop BPD^69^. However, this report does not investigate sex-specific differences in MMP-3 levels. It is also possible that regional differences in MMP-3 levels specific to the niche of lung mesenchymal cells exist and influence extracellular matrix remodeling. Based on the present and the above prior studies we propose that defective PDGFRα and WNT2 signaling is a primary feature of BPD development in male premature infants with RDS.

Since our study identifies male sex-specific gene expression differences in lung MSC from infants developing BPD, it is important to consider a role for sex steroid signaling. Testosterone, produced by fetal testes following sex differentiation^70^, modulates fetal lung fibroblast synthetic function^71^, delays surfactant production during mid-late gestation^72^ and thus may contribute to RDS severity. Estrogen receptors, expressed in the fetal lung^73,74^, promote lung maturation^75^ and favor alveolar formation in females in murine studies^76,77^. The effects of sex steroids on lung mesenchymal cells have not been investigated and will be the focus of our future studies.

Lung resident MSCs have been isolated from bronchoalveolar lavage fluid from patients with other lung diseases resultant from aberrant injury and repair, e.g. lung transplant recipients and idiopathic pulmonary fibrosis patients^78^. Gene expression differences in lung resident MSCs from patients with idiopathic pulmonary fibrosis, a disease marked by myofibroblast accumulation and extracellular matrix deposition, distinguish between progressive and stable phenotypes^79^. These studies provide support that, despite the influence of *ex vivo* cell culture conditions, gene expression differences are preserved and transcriptomic profiling of lung resident MSCs may be used to identify disease phenotypes. Consistent with this, the transcriptomic differences identified in the discovery cohort of our study were confirmed in MSCs from a second, larger cohort of premature infants, suggesting that MSCs from tracheal aspirates of preterm infants with RDS maintain a stable pattern of gene expression.

Unlike MSCs from male infants developing BPD, MSCs from female infants developing BPD did not show distinct gene expression differences compared to MSCs from both male and female infants who were not developing BPD. To further define the mechanisms of BPD development in female infants, we focused on the role of inflammation and infection. Chorioamnionitis and sepsis are risk factors for BPD development^41,42^. Furthermore, female premature infants exposed to chorioamnionitis have significantly lower lung function than those not exposed, an effect not observed in male infants^80^, suggesting that female infants may be more susceptible to the effects of chorioamnionitis than male infants. Interestingly, in animal studies, hyperoxic exposure of immature mice induces a greater increase in CCL2 in female compared to male neonatal mice^16^, indicating a propensity for greater CCL2 responses in females compared to males. Our findings that tracheal aspirate CCL2 levels positively correlated with Gal-1 levels and CCL2 and Gal-1 levels were significantly higher in tracheal aspirates from female infants developing BPD compared to female infants who were not developing BPD and male infants developing BPD may reflect the higher incidence of chorioamnionitis among female infants developing BPD in our cohort. Nevertheless, these results highlight the need for broader phenotyping of the inflammatory environment in the airways of female and male premature infants with RDS.

There are several limitations to our study. First, examining the cellular or molecular components of tracheal aspirates may not reflect processes in the distal lung. However, previous studies have shown neonatal tracheal aspirate fluid to have equal validity to bronchoalveolar lavage in the estimation of disaturated phosphatidylcholine^81^, IL-8 levels and percentage of polymorphonuclear cells^82^, and therefore these aspirates are a practical alternative for obtaining neonatal lung fluid specimens. Second, it is possible that the lung MSCs isolated from male infants developing BPD represent a different population of cells that are recruited to the airway lumen, therefore explaining differences in gene expression. However, one would expect that a recruited cell population would be specific to outcome-only or sex-only, but not to both. Third, we are not able to distinguish whether the distinctive gene signature in MSCs from male infants developing BPD is due to sex-specific developmental differences in gene expression or due to sex-related differential response to perinatal exposures. Fourth, the MSCs were expanded *in vitro* prior to analysis; to minimize the possibility that cells starting off with a different phenotype may have nonuniform expansion, all cells were cultured under the same conditions and monitored and achieved similar confluence levels within similar timeframe. Therefore, we believe that the *in vitro* cell culture effect was minimized and the distinctive gene expression signature of MSCs from male infants developing BPD reflects inherent transcriptional differences. Fifth, our study is limited by the demographic characteristics of the patients studied. Our analysis only included premature infants, who were receiving mechanical ventilation for RDS in the first week of life and the majority of the patients were Caucasian. Future studies involving larger cohorts of racially and ethnically diverse patients are needed to validate our results. Nevertheless, we believe our study is the first one to unveil the heterogeneous phenotype of human BPD.

In conclusion, our results support the notion of sex-specific differences in the cellular and molecular mechanisms of neonatal RDS in premature infants that favor BPD development. Defining the sex-specific phenotypes based on cellular and molecular markers in the first week of life may help identify targeted therapies for prevention or treatment of BPD.

## Methods

### Patients

We collected tracheal aspirates from infants admitted to the University of Michigan C.S. Mott Children’s Hospital Newborn ICU. Entry criteria included gestational age at birth of <32 weeks, mechanical ventilation for respiratory distress, and age <7 days. Infants with acute sepsis during their first week of life were excluded. Chorioamnionitis and necrotizing enterocolitis were diagnosed clinically. The study was approved by the University of Michigan Medical School Institutional Review Board. Written informed consent was obtained from both parents. All methods involving human participants were performed in accordance with the relevant guidelines and regulations.

### Tracheal aspirate collection and MSC isolation

The endotracheal tube was suctioned as needed to maintain tube patency. The need for suctioning was determined by the nurse or respiratory therapist. MSCs were isolated from tracheal aspirates as described previously^34^. Specimens were centrifuged at 1,200 g for 5 minutes at 15 °C and supernatants were stored at −80 °C. MSCs of low passage number (2 to 4) were studied.

### Cell culture and RNA preparation

Cells were maintained in 10% MSC fetal bovine serum, 1% penicillin-streptomycin, 1% L-glutamine, and 0.5% amphotericin B in a 100-mm plate until 70–90% confluent. Adherent cells were incubated at 37 °C and 5% CO2. The cells were serum starved for 4 hours prior to harvest to minimize differences in cell cycle related gene expression. Total RNA was extracted using RNeasy Plus Mini kit (Qiagen, Valencia, CA).

### Gene array

We examined the gene expression profile of 13 individual neonatal lung MSC isolates using the Illumina HumanRefSeq-8v3 expression BeadChip platform (San Diego, CA). This system covers >24,000 probes for >18,000 unique sequences from the NCBI RefSeq database. The analysis was carried out by the University of Michigan Sequencing Core. Hybridized biotinylated cRNA was detected with streptavidin-Cy3 and quantified using the Illumina Bead Array Reader.

### Microarray analysis

For microarray analysis data were extracted using GenomeStudio Data Analysis Software (Illumina) and quantile normalization was performed. Genes with low mean expression (less than 100) and variance less than 2 standard deviations outside of the mean were filtered out. Multivariate data analysis, including exploratory cluster analysis was performed using R-software 3.2.2.

### Differential gene expression and pathway enrichment analysis

We compared gene expression differences between the two main clusters identified by the exploratory multivariate data analysis - male infants developing BPD and a Combined Control group. The Combined Control group comprised of male infants who did not go on to develop BPD and all female infants. Significant difference in gene expression was defined by unpaired *t*-test and P less than 0.05 and fold change greater than or equal to 2. Differentially expressed genes were analyzed for over-representation of pathways and gene ontology terms via the meta-database ConcensusPathDB (http://cpdb.molgen.mpg.de/)^38,39^.

### Quantitative real-time PCR

The expression of selected genes was quantified with SYBR Green technology. The specific primer sequences are available upon request. Relative gene expression was analyzed with the 2^−ΔCT^ algorithm by normalizing the level of gene expression for each sample to glyceraldehyde 3-phosphate dehydrogenase (GAPDH).

### Immunoblotting

MSC lysates were resolved by SDS-PAGE, and transferred to a nitrocellulose membrane. Membranes were blocked in 5% milk for 1 h in room temperature and probed with antibodies against PDGFR-α (Cell Signaling, Danvers, MA).

### Measurement of FGF7, Wnt2, MMP3, CCL2 and Galectin-1 protein levels

Protein levels of FGF7, Wnt2 and MMP3 in MSC supernatants were measured by enzyme-linked immunosorbent assay (FGF7 and MMP3 from R&D Systems, Minneapolis, MN and Wnt2 from CUSABIO Biotech, China). Tracheal aspirate protein levels of CCL2 were measured by multiplex assay (Bio-Rad, Hercules, CA) and Galectin-1 levels were measure by enzyme-linked immunosorbent assay (R&D Systems).

### Statistical analysis

Data were described as means ± SEM. Statistical significance was assessed by unpaired *t-*test or one-way ANOVA, as appropriate. *P* values were considered statistically significant if they were <0.05.

## Electronic supplementary material

Supplementary information

## Data Availability

Microarray data will be available from the NCBI Gene Expression Omnibus (GEO) upon publication of the manuscript.
