# Supplementary material for: Gene Expression Signatures Point to a Male Sex-Specific Lung Mesenchymal Cell PDGF Receptor Signaling Defect in Infants Developing Bronchopulmonary Dysplasia
Source: Sci Rep. 2018 Nov 20;8:17070. doi: 10.1038/s41598-018-35256-z (PMC6244280; doi:10.1038/s41598-018-35256-z)
Supplement: Supplementary file 1 — Supplementary information [file 41598_2018_35256_MOESM1_ESM.pdf]

Gene Expression Signatures Point to a Male Sex-Specific Lung Mesenchymal Cell PDGF Receptor Signaling Defect in Infants Developing Bronchopulmonary Dysplasia

Christina T. Fulton[1], Tracy X. Cui[1], Adam M. Goldsmith[1], Jennifer Bermick[2], Antonia P. Popova[1*]

[1]Division of Pediatric Pulmonology, [2]Division of Neonatal-Perinatal Medicine, Department of Pediatrics and Communicable Diseases, University of Michigan, Ann Arbor, MI USA.

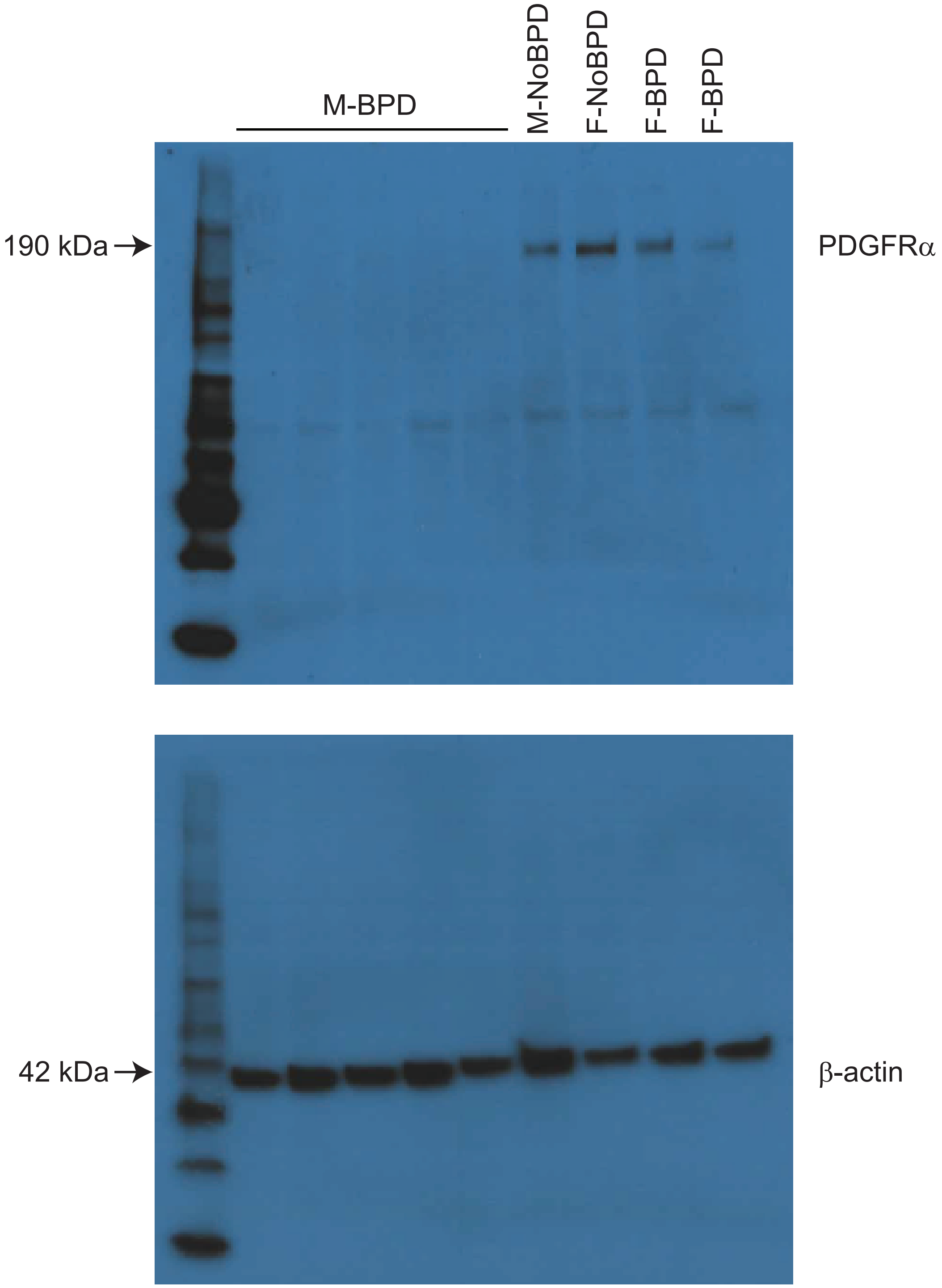

Supplemental Figure S1. Decreased PDGFRα protein expression in MSCs from male infants developing BPD. Cell lysates from MSCs from 5 male infants developing BPD and 4 infants from the Combined control group (one male and one female infants who were not developing BPD and two female infants developing BPD) were prepared in RIPA buffer and resolved by SDS-PAGE, and transferred to a nitrocellulose membrane. Membranes were blocked in 5% milk for 1h in room temperature and probed with antibodies against PDGFR-α; β-actin antibody was used as loading control.

Supplemental Table S1. Differentially regulated genes between M-BPD and Combined control MSCs.

Symbol	Name	Entrez Gene	*P* Value	Fold Change
WNT2	wingless-type MMTV integration site family member 2	7472	4.68E-03	0.05790922
ADH1A	alcohol dehydrogenase 1A (class I), alpha polypeptide	124	3.00E-02	0.062756606
MASP1	mannan-binding lectin serine peptidase 1 (C4/C2 activating component of Ra-reactive factor) (MASP1), transcript variant 2	5648	2.78E-03	0.090760835
WFDC1	WAP four-disulfide core domain 1	58189	1.77E-03	0.093876979
PTGS1	prostaglandin-endoperoxide synthase 1 (prostaglandin G/H synthase and cyclooxygenase) (PTGS1), transcript variant 1	5742	2.44E-03	0.097542469
PITX1	paired-like homeodomain transcription factor 1	5307	5.67E-04	0.099215153
TFAP2C	transcription factor AP-2 gamma (activating enhancer binding protein 2 gamma)	7022	1.71E-02	0.111240595
C1orf115	chromosome 1 open reading frame 115	79762	1.14E-02	0.129585776
RASL11B	RAS-like, family 11, member B	65997	2.50E-02	0.133074705
BMP4	bone morphogenetic protein 4 (BMP4), transcript variant 1	652	1.52E-02	0.133783272
DLL1	delta-like 1 (Drosophila)	28514	1.98E-02	0.142291306
ADH1B	alcohol dehydrogenase IB (class I), beta polypeptide	125	3.74E-02	0.142522179
SEMA4D	sema domain, immunoglobulin domain (Ig), transmembrane domain (TM) and short cytoplasmic domain, (semaphorin) 4D	10507	5.91E-03	0.148240379
GLDN	gliomedin	342035	1.17E-02	0.151179871
MMP3	matrix metallopeptidase 3 (stromelysin 1, progelatinase)	4314	8.90E-03	0.156368127
RASL12	RAS-like, family 12	51285	1.22E-02	0.159033926
CXCL12	chemokine (C-X-C motif) ligand 12 (stromal cell-derived factor 1)	6387	1.31E-02	0.164946061
CTSC	cathepsin C (CTSC), transcript variant 1	1075	1.51E-03	0.174988714
FOXQ1	forkhead box Q1	94234	1.56E-02	0.180328564
RASSF2	Ras association (RalGDS/AF-6) domain family member 2 (RASSF2), transcript variant 2	9770	5.22E-03	0.18052733
MXRA5	matrix-remodelling associated 5	25878	1.89E-02	0.184090117
SPRY1	sprouty homolog 1, antagonist of FGF signaling (Drosophila)	10252	3.31E-03	0.184654435
ADAMTS1	ADAM metallopeptidase with thrombospondin type 1 motif, 1	9510	1.99E-03	0.185496801
RASL11A	RAS-like, family 11, member A	387496	3.07E-02	0.191153755
TEK	TEK tyrosine kinase, endothelial (venous malformations, multiple cutaneous and mucosal)	7010	1.05E-02	0.191317628
ADAMTS8	ADAM metallopeptidase with thrombospondin type 1 motif, 8	11095	1.30E-02	0.194763324

Supplemental Table S1. Differentially regulated genes between M-BPD and Combined control MSCs.

ADAMTS5	ADAM metallopeptidase with thrombospondin type 1 motif, 5 (aggrecanase-2)	11096	2.95E-03	0.195290323
LEF1	lymphoid enhancer-binding factor 1	51176	2.77E-02	0.210406598
PTH1R	parathyroid hormone 1 receptor	5745	6.81E-03	0.215773643
TMTC1	transmembrane and tetratricopeptide repeat containing 1	83857	8.30E-04	0.217721193
D4S234E	DNA segment on chromosome 4 (unique) 234 expressed sequence (D4S234E), transcript variant 2	27065	3.64E-02	0.219843807
RGS5	regulator of G-protein signaling 5	8490	1.29E-02	0.221424941
TCF21	transcription factor 21 (TCF21), transcript variant 1	6943	1.02E-03	0.222458659
ITGA8	integrin, alpha 8	8516	4.35E-02	0.224354186
GDF10	growth differentiation factor 10	2662	4.85E-02	0.225983137
TSPAN12	tetraspanin 12	23554	4.51E-02	0.23029638
HBG2	hemoglobin, gamma G	3048	2.31E-02	0.238372093
PSG4	pregnancy specific beta-1-glycoprotein 4 (PSG4), transcript variant 1	5672	1.28E-02	0.238714146
COL4A5	collagen, type IV, alpha 5 (COL4A5), transcript variant 1	1287	3.08E-02	0.239301649
PDGFD	platelet derived growth factor D (PDGFD), transcript variant 2	80310	1.86E-03	0.245952473
CXCR7	chemokine (C-X-C motif) receptor 7	57007	2.77E-02	0.254071276
C13orf16	chromosome 13 open reading frame 16	121793	9.15E-03	0.259348637
HBG1	hemoglobin, gamma A	3047	2.40E-02	0.261075949
SVEP1	sushi, von Willebrand factor type A, EGF and pentraxin domain containing 1	79987	3.49E-02	0.274755837
KCNF1	potassium voltage-gated channel, subfamily F, member 1	3754	3.26E-02	0.279419578
CLDN23	claudin 23	137075	1.11E-02	0.281102914
TRPA1	transient receptor potential cation channel, subfamily A, member 1	8989	3.49E-02	0.281321249
FOXF2	forkhead box F2	2295	1.05E-03	0.281773302
PTGER2	prostaglandin E receptor 2 (subtype EP2), 53kDa	5732	6.43E-03	0.281785477
C13orf15	chromosome 13 open reading frame 15	28984	1.43E-02	0.283599102
CYGB	cytoglobin	114757	2.67E-02	0.284965695
ALPL	alkaline phosphatase, liver/bone/kidney (ALPL), transcript variant 1	249	2.22E-02	0.288576737
FGF7	fibroblast growth factor 7 (keratinocyte growth factor)	2252	4.23E-03	0.289487871
ANGPTL2	angiopoietin-like 2	23452	4.28E-03	0.295872802
STOM	stomatin (STOM), transcript variant 1	2040	4.00E-04	0.297940966
SNCAIP	synuclein, alpha interacting protein	9627	4.11E-02	0.299504817
PSG7	pregnancy specific beta-1-glycoprotein 7	5676	1.60E-02	0.301350646
PCDH18	protocadherin 18	54510	1.08E-03	0.302916749
FHL1	four and a half LIM domains 1	2273	1.17E-03	0.30767727
PPAP2A	phosphatidic acid phosphatase type 2A (PPAP2A), transcript variant 1	8611	9.81E-03	0.308210713
PDE7B	phosphodiesterase 7B	27115	6.76E-03	0.308299578

Supplemental Table S1. Differentially regulated genes between M-BPD and Combined control MSCs.

C10orf10	chromosome 10 open reading frame 10	11067	1.50E-02	0.309082556
SLC16A6	solute carrier family 16, member 6 (monocarboxylic acid transporter 7)	9120	8.76E-03	0.311986648
COLEC12	collectin sub-family member 12	81035	2.52E-02	0.314035647
TGFBR3	transforming growth factor, beta receptor III	7049	1.01E-02	0.314138057
SMAD9	SMAD family member 9	4093	2.65E-03	0.314952917
NR2F1	nuclear receptor subfamily 2, group F, member 1	7025	2.18E-03	0.316649408
PTGES	prostaglandin E synthase	9536	1.47E-02	0.320290092
GPR44	G protein-coupled receptor 44	11251	3.95E-02	0.323620309
RDH10	retinol dehydrogenase 10 (all-trans)	157506	1.01E-02	0.324372535
SMPDL3A	sphingomyelin phosphodiesterase, acid-like 3A	10924	2.80E-02	0.325068386
ETS2	v-ets erythroblastosis virus E26 oncogene homolog 2 (avian)	2114	6.27E-03	0.329525989
C10orf54	chromosome 10 open reading frame 54	64115	4.73E-03	0.329922605
SLC40A1	solute carrier family 40 (iron-regulated transporter), member 1	30061	8.47E-03	0.332963035
TRPC6	transient receptor potential cation channel, subfamily C, member 6	7225	5.98E-04	0.333003537
PAG1	phosphoprotein associated with glycosphingolipid microdomains 1	55824	1.12E-02	0.336073672
SLC16A14	solute carrier family 16, member 14 (monocarboxylic acid transporter 14)	151473	1.88E-02	0.33678312
RARRES2	retinoic acid receptor responder (tazarotene induced) 2	5919	2.19E-02	0.339150651
SERPINF1	serpin peptidase inhibitor, clade F (alpha-2 antiplasmin, pigment epithelium derived factor), member 1	5176	2.16E-02	0.339298207
FAM20C	family with sequence similarity 20, member C	56975	3.43E-03	0.345193765
ASS1	argininosuccinate synthetase 1 (ASS1), transcript variant 1	445	1.23E-02	0.346559053
ALG1L	asparagine-linked glycosylation 1-like	200810	5.41E-03	0.347210912
FAM46C	family with sequence similarity 46, member C	54855	3.14E-02	0.347357269
BHMT2	betaine-homocysteine methyltransferase 2	23743	7.11E-03	0.348503212
FGF9	fibroblast growth factor 9 (glia-activating factor)	2254	1.76E-03	0.348920162
CRYBB2	crystallin, beta B2	1415	4.29E-02	0.351649526
ATP8B4	ATPase, class I, type 8B, member 4	79895	2.62E-02	0.352810429
SLC2A12	solute carrier family 2 (facilitated glucose transporter), member 12	154091	1.60E-02	0.356095466
GFRA1	GDNF family receptor alpha 1 (GFRA1), transcript variant 1	2674	1.00E-02	0.360925339
PPAPDC3	phosphatidic acid phosphatase type 2 domain containing 3	84814	3.89E-02	0.361092695
ZNF608	zinc finger protein 608	57507	9.36E-03	0.363341724
STOX2	storkhead box 2	56977	1.51E-02	0.363641629
COL21A1	collagen, type XXI, alpha 1	81578	4.52E-02	0.364224138
PLD1	phospholipase D1, phosphatidylcholine-specific	5337	7.75E-04	0.364812442

Supplemental Table S1. Differentially regulated genes between M-BPD and Combined control MSCs.

FHOD3	formin homology 2 domain containing 3	80206	1.03E-02	0.368964862
RECK	reversion-inducing-cysteine-rich protein with kazal motifs	8434	1.42E-02	0.369402985
PODN	podocan	127435	1.17E-02	0.378856949
SLC9A9	solute carrier family 9 (sodium/hydrogen exchanger), member 9	285195	9.14E-03	0.379398143
LTBP4	latent transforming growth factor beta binding protein 4 (LTBP4), transcript variant 2	8425	1.64E-02	0.379989765
TOX	thymocyte selection-associated high mobility group box	9760	2.03E-03	0.380882781
FBN2	fibrillin 2	2201	3.80E-03	0.381511514
DACH1	dachshund homolog 1 (Drosophila)	1602	4.55E-02	0.383723591
C10orf107	chromosome 10 open reading frame 107	219621	9.64E-04	0.385732997
SDPR	serum deprivation response (phosphatidylserine binding protein)	8436	1.58E-02	0.39001627
OLFML2A	olfactomedin-like 2A	169611	3.12E-02	0.392224727
IL1R1	interleukin 1 receptor, type I	3554	3.29E-02	0.394620519
MATN2	matrilin 2 (MATN2), transcript variant 1	4147	1.66E-02	0.39466436
KLHL13	kelch-like 13 (Drosophila)	90293	2.36E-02	0.396594512
RAB38	RAB38, member RAS oncogene family	23682	4.57E-03	0.398813612
SSH2	slingshot homolog 2 (Drosophila)	85464	3.94E-03	0.400186668
CES1	carboxylesterase 1 (monocyte/macrophage serine esterase 1) (CES1), transcript variant 1	1066	3.53E-02	0.412398799
PDE5A	phosphodiesterase 5A, cGMP-specific (PDE5A), transcript variant 1	8654	9.11E-03	0.414949365
LRP1B	low density lipoprotein-related protein 1B (deleted in tumors)	53353	1.80E-02	0.417679942
OR7E37P	olfactory receptor, family 7, subfamily E, member 37 pseudogene	26636	4.81E-03	0.418416651
GSTM3	glutathione S-transferase M3 (brain)	2947	3.14E-02	0.420413037
PLXNC1	plexin C1	10154	5.35E-03	0.42144205
DENND2A	DENN/MADD domain containing 2A	27147	2.10E-02	0.421611449
VWA5A	von Willebrand factor A domain containing 5A	4013	1.17E-02	0.422566834
NPC1L1	NPC1 (Niemann-Pick disease, type C1, gene)-like 1	29881	1.07E-02	0.426324115
PDGFRA	platelet-derived growth factor receptor, alpha polypeptide	5156	1.86E-02	0.430744052
VGLL3	vestigial like 3 (Drosophila)	389136	7.91E-03	0.431436534
PTGR1	prostaglandin reductase 1	22949	2.66E-02	0.431482634
ST3GAL1	ST3 beta-galactoside alpha-2,3-sialyltransferase 1 (ST3GAL1), transcript variant 1	6482	2.15E-02	0.432400558
EPHB6	EPH receptor B6	2051	7.46E-03	0.434034239
C6orf192	chromosome 6 open reading frame 192	116843	9.28E-03	0.435030021
PLEKHA5	pleckstrin homology domain containing, family A member 5	54477	4.96E-03	0.435205132
RNASE4	ribonuclease, RNase A family, 4 (RNASE4), transcript variant 3	6038	1.31E-02	0.437502358
CORIN	corin, serine peptidase	10699	3.22E-02	0.44477879

Supplemental Table S1. Differentially regulated genes between M-BPD and Combined control MSCs.

MYLIP	myosin regulatory light chain interacting protein	29116	1.67E-02	0.445791667
SGCD	sarcoglycan, delta (35kDa dystrophin-associated glycoprotein)	6444	1.27E-02	0.446646046
GZMH	granzyme H (cathepsin G-like 2, protein h-CCPX)	2999	7.79E-03	0.447917646
NFIA	nuclear factor I/A	4774	6.09E-03	0.462050437
SEMA5A	sema domain, seven thrombospondin repeats (type 1 and type 1-like), transmembrane domain (TM) and short cytoplasmic domain, (semaphorin) 5A	9037	3.29E-02	0.464738774
RGS2	regulator of G-protein signalling 2, 24kDa	5997	3.16E-02	0.466818298
FAIM3	Fas apoptotic inhibitory molecule 3	9214	2.74E-02	0.467104868
PRICKLE1	prickle homolog 1 (Drosophila)	144165	3.86E-02	0.468179891
EDG1	endothelial differentiation, sphingolipid G-protein-coupled receptor, 1	1901	2.55E-02	0.468417892
TWIST1	twist homolog 1 (Drosophila)	7291	2.59E-02	0.472773939
MMP27	matrix metallopeptidase 27	64066	2.66E-02	0.473469814
KITLG	KIT ligand (KITLG), transcript variant b	4254	1.97E-02	0.482582029
FBLN1	fibulin 1 (FBLN1), transcript variant A	2192	3.45E-02	0.486247449
AGPAT9	1-acylglycerol-3-phosphate O-acyltransferase 9	84803	2.48E-02	0.489328099
NID1	nidogen 1	4811	1.76E-02	0.490661616
MCOLN2	mucolipin 2	255231	1.45E-02	0.494251672
TPM1	tropomyosin 1 (alpha) (TPM1), transcript variant 3	7168	4.99E-02	2.108024216
CCND1	cyclin D1	595	3.64E-02	2.213577643
PLXDC2	plexin domain containing 2	84898	2.73E-02	2.270855633
NP	nucleoside phosphorylase	4860	1.47E-02	2.44724026
ZFY	zinc finger protein, Y-linked	7544	1.38E-02	2.543190416
ETV5	ets variant gene 5 (ets-related molecule)	2119	3.27E-06	2.586875331
TNFRSF12A	tumor necrosis factor receptor superfamily, member 12A	51330	5.87E-03	2.67104826
JARID1D	jumonji, AT rich interactive domain 1D	8284	6.48E-04	2.704488778
ARHGAP22	Rho GTPase activating protein 22	58504	9.43E-04	2.764136833
GSTO2	glutathione S-transferase omega 2	119391	3.94E-03	2.90846483
NLGN4Y	neuroligin 4, Y-linked	22829	2.92E-03	2.954520397
SLC20A1	solute carrier family 20 (phosphate transporter), member 1	6574	2.54E-02	2.971969631
CDH2	cadherin 2, type 1, N-cadherin (neuronal)	1000	2.03E-02	3.011740447
CYorf15A	chromosome Y open reading frame 15A	246126	8.86E-05	3.030498949
SEMA3C	sema domain, immunoglobulin domain (Ig), short basic domain, secreted, (semaphorin) 3C	10512	3.66E-02	3.045604698
FLJ14213	protor-2	79899	2.30E-02	3.133914662
EIF1AY	eukaryotic translation initiation factor 1A, Y-linked	9086	2.45E-04	3.186420722
ODZ1	odz, odd Oz/ten-m homolog 1(Drosophila)	10178	2.36E-02	3.548699832
RPS4Y2	ribosomal protein S4, Y-linked 2	140032	6.22E-04	3.654638806
BCHE	butyrylcholinesterase	590	1.20E-02	4.102194925

Supplemental Table S1. Differentially regulated genes between M-BPD and Combined control MSCs.

IL1A	interleukin 1, alpha	3552	3.18E-02	4.468812438
RPS4Y1	ribosomal protein S4, Y-linked 1	6192	3.85E-04	4.574757925

Supplemental Table S2. Enriched pathway-based sets.

pathway	source	external_id	members_input_overlap	members_input_overlap_geneids	p-value	q-value
Extracellular matrix organization	Reactome	R-HSA-1474244	ITGA8; ADAMTS5; BMP4; ADAMTS1; COL4A5; FBN2; ADAMTS8; NID1; LTBP4; COL21A1; FBLN1; MMP3	8516; 2192; 4811; 11095; 652; 2201; 4314; 8425; 11096; 81578; 1287; 9510	1.00E-05	1.49E-03
Elastic fibre formation	Reactome	R-HSA-1566948	ITGA8; BMP4; FBLN1; LTBP4; FBN2	2201; 2192; 8516; 8425; 652	1.25E-05	1.49E-03
Molecules associated with elastic fibres	Reactome	R-HSA-2129379	ITGA8; BMP4; FBLN1; LTBP4	2192; 8516; 8425; 652	1.29E-04	8.77E-03
Axon guidance - Homo sapiens (human)	KEGG	path:hsa04360	PLXNC1; SEMA3C; EPHB6; SEMA5A; TRPC6; SSH2; CXCL12; SEMA4D	2051; 7225; 6387; 10507; 10512; 9037; 10154; 85464	1.56E-04	8.77E-03
Pathways in cancer - Homo sapiens (human)	KEGG	path:hsa05200	PDGFRA; BMP4; PLD1; CCND1; WNT2; PTGER2; COL4A5; FGF9; FGF7; KITLG; CXCL12; LEF1	4254; 2252; 2254; 6387; 595; 652; 5156; 5337; 1287; 7472; 5732; 51176	1.84E-04	8.77E-03
O-glycosylation of TSR domain-containing proteins	Reactome	R-HSA-5173214	ADAMTS5; ADAMTS1; ADAMTS8; SEMA5A	11096; 11095; 9037; 9510	3.64E-04	1.32E-02
Melanoma - Homo sapiens (human)	KEGG	path:hsa05218	FGF7; CCND1; PDGFRA; PDGFD; FGF9	5156; 80310; 2252; 2254; 595	3.90E-04	1.32E-02
Other semaphorin interactions	Reactome	R-HSA-416700	PLXNC1; SEMA4D; SEMA5A	9037; 10154; 10507	5.74E-04	1.71E-02
Ras signaling pathway - Homo sapiens (human)	KEGG	path:hsa04014	PDGFRA; TEK; PDGFD; PLD1; ETS2; FGF9; FGF7; KITLG	5156; 2114; 4254; 2252; 2254; 5337; 7010; 80310	8.82E-04	2.33E-02
eicosanoid metabolism	BioCarta	eicosanoidpathway	PTGS1; PTGES; PTGER2	5742; 9536; 5732	1.02E-03	2.44E-02
TRP channels	Reactome	R-HSA-3295583	TRPA1; TRPC6; MCOLN2	8989; 7225; 255231	1.31E-03	2.84E-02
Breast cancer - Homo sapiens (human)	KEGG	path:hsa05224	CCND1; WNT2; DLL1; FGF9; FGF7; LEF1	595; 2252; 2254; 7472; 28514; 51176	1.78E-03	3.54E-02
Degradation of the extracellular matrix	Reactome	R-HSA-1474228	ADAMTS5; NID1; ADAMTS1; ADAMTS8; MMP3	4811; 11095; 4314; 11096; 9510	2.48E-03	4.28E-02
Constitutive Signaling by Aberrant PI3K in Cancer	Reactome	R-HSA-2219530	FGF7; KITLG; PDGFRA; FGF9	4254; 2254; 5156; 2252	2.52E-03	4.28E-02
PI3K-Akt signaling pathway - Homo sapiens (human)	KEGG	path:hsa04151	ITGA8; PDGFRA; TEK; PDGFD; CCND1; COL4A5; FGF9; FGF7; KITLG	5156; 4254; 2252; 2254; 1287; 8516; 7010; 80310; 595	3.00E-03	4.44E-02
Drug metabolism - cytochrome P450 - Homo sapiens (human)	KEGG	path:hsa00982	GSTO2; GSTM3; ADH1B; ADH1A	2947; 119391; 124; 125	3.13E-03	4.44E-02
Axon guidance	Reactome	R-HSA-422475	PLXNC1; PDGFRA; TEK; EPHB6; SEMA5A; COL4A5; GFRA1; FGF9; FGF7; KITLG; SEMA4D	1287; 5156; 2252; 9037; 4254; 2254; 10154; 7010; 2051; 10507; 2674	3.45E-03	4.44E-02
O-linked glycosylation	Reactome	R-HSA-5173105	ADAMTS5; ST3GAL1; ADAMTS1; ADAMTS8; SEMA5A	9037; 11095; 11096; 6482; 9510	3.65E-03	4.44E-02

Arrhythmogenic right ventricular cardiomyopathy (ARVC) - Homo sapiens (human)	KEGG	path:hsa05412	ITGA8; CDH2; LEF1; SGCD	6444; 8516; 1000; 51176	3.65E-03	4.44E-02
Metabolism of xenobiotics by cytochrome P450 - Homo sapiens (human)	KEGG	path:hsa00980	GSTO2; GSTM3; ADH1B; ADH1A	2947; 124; 119391; 125	3.83E-03	4.44E-02
regulators of bone mineralization	BioCarta	npp1pathway	COL4A5; ALPL	1287; 249	3.98E-03	4.44E-02
NCAM signaling for neurite out-growth	Reactome	R-HSA-375165	PDGFRA; TEK; COL4A5; GFRA1; FGF9; FGF7; KITLG	1287; 4254; 2674; 2254; 7010; 2252; 5156	4.10E-03	4.44E-02
Ethanol oxidation	Reactome	R-HSA-71384	ADH1B; ADH1A	124; 125	4.75E-03	4.92E-02
GAB1 signalosome	Reactome	R-HSA-180292	FGF7; PAG1; KITLG; PDGFRA; FGF9	4254; 2254; 2252; 55824; 5156	5.34E-03	4.93E-02
Chemical carcinogenesis - Homo sapiens (human)	KEGG	path:hsa05204	GSTO2; GSTM3; ADH1B; ADH1A	2947; 124; 119391; 125	5.80E-03	4.93E-02
Signaling by EGFR	Reactome	R-HSA-177929	PDGFRA; TEK; PAG1; SPRY1; GFRA1; FGF9; FGF7; KITLG	4254; 55824; 2674; 10252; 7010; 2254; 2252; 5156	6.85E-03	4.93E-02
PI5P, PP2A and IER3 Regulate PI3K/AKT Signaling	Reactome	R-HSA-6811558	FGF7; KITLG; PDGFRA; FGF9	4254; 2254; 2252; 5156	7.14E-03	4.93E-02
Rheumatoid arthritis - Homo sapiens (human)	KEGG	path:hsa05323	TEK; CXCL12; MMP3; IL1A	4314; 6387; 7010; 3552	7.73E-03	4.93E-02
Prostate cancer - Homo sapiens (human)	KEGG	path:hsa05215	CCND1; PDGFRA; PDGFD; LEF1	5156; 595; 80310; 51176	7.73E-03	4.93E-02
PI3K/AKT Signaling in Cancer	Reactome	R-HSA-2219528	FGF7; KITLG; PDGFRA; FGF9	4254; 2254; 2252; 5156	8.04E-03	4.93E-02
RAF/MAP kinase cascade	Reactome	R-HSA-5673001	PDGFRA; TEK; GFRA1; FGF9; FGF7; KITLG	4254; 2674; 2254; 7010; 2252; 5156	8.11E-03	4.93E-02
SHC1 events in EGFR signaling	Reactome	R-HSA-180336	PDGFRA; TEK; GFRA1; FGF9; FGF7; KITLG	4254; 2674; 2254; 7010; 2252; 5156	8.11E-03	4.93E-02
SOS-mediated signalling	Reactome	R-HSA-112412	PDGFRA; TEK; GFRA1; FGF9; FGF7; KITLG	4254; 2674; 2254; 7010; 2252; 5156	8.11E-03	4.93E-02
GRB2 events in EGFR signaling	Reactome	R-HSA-179812	PDGFRA; TEK; GFRA1; FGF9; FGF7; KITLG	4254; 2674; 2254; 7010; 2252; 5156	8.11E-03	4.93E-02
Wnt signaling pathway - Homo sapiens (human)	KEGG	path:hsa04310	CCND1; PRICKLE1; LEF1; SERPINF1; WNT2	5176; 595; 144165; 7472; 51176	8.22E-03	4.93E-02
Gastrin-CREB signalling pathway via PKC and MAPK	Reactome	R-HSA-881907	PDGFRA; TEK; RGS2; TRPC6; FGF9; FGF7; GFRA1; KITLG; MMP3	4254; 2252; 5997; 2254; 7225; 7010; 5156; 2674; 4314	8.39E-03	4.93E-02
Synthesis of Prostaglandins (PG) and Thromboxanes (TX)	Reactome	R-HSA-2162123	PTGS1; PTGES	5742; 9536	8.44E-03	4.93E-02
Signaling by PDGF	Reactome	R-HSA-186797	PDGFRA; TEK; PDGFD; COL4A5; GFRA1; FGF9; FGF7; KITLG	4254; 2674; 7010; 2254; 2252; 80310; 1287; 5156	8.49E-03	4.93E-02
Signalling to p38 via RIT and RIN	Reactome	R-HSA-187706	PDGFRA; TEK; GFRA1; FGF9; FGF7; KITLG	4254; 2674; 2254; 7010; 2252; 5156	8.90E-03	4.93E-02
ARMS-mediated activation	Reactome	R-HSA-170984	PDGFRA; TEK; GFRA1; FGF9; FGF7; KITLG	4254; 2674; 2254; 7010; 2252; 5156	8.90E-03	4.93E-02
Frs2-mediated activation	Reactome	R-HSA-170968	PDGFRA; TEK; GFRA1; FGF9; FGF7; KITLG	4254; 2674; 2254; 7010; 2252; 5156	9.11E-03	4.93E-02

MAPK1/MAPK3 signaling	Reactome	R-HSA-5684996	PDGFRA; TEK; GFRA1; FGF9; FGF7; KITLG	4254; 2674; 2254; 7010; 2252; 5156	9.31E-03	4.93E-02
Negative regulation of the PI3K/AKT network	Reactome	R-HSA-199418	FGF7; KITLG; PDGFRA; FGF9	4254; 2254; 2252; 5156	9.34E-03	4.93E-02
Activated point mutants of FGFR2	Reactome	R-HSA-2033519	FGF7; FGF9	2254; 2252	9.52E-03	4.93E-02
FGFR2 mutant receptor activation	Reactome	R-HSA-1839126	FGF7; FGF9	2254; 2252	9.52E-03	4.93E-02
Prolonged ERK activation events	Reactome	R-HSA-169893	PDGFRA; TEK; GFRA1; FGF9; FGF7; KITLG	4254; 2674; 2254; 7010; 2252; 5156	9.53E-03	4.93E-02
Signaling by Leptin	Reactome	R-HSA-2586552	PDGFRA; TEK; GFRA1; FGF9; FGF7; KITLG	4254; 2674; 2254; 7010; 2252; 5156	9.74E-03	4.93E-02
Signalling to RAS	Reactome	R-HSA-167044	PDGFRA; TEK; GFRA1; FGF9; FGF7; KITLG	4254; 2674; 2254; 7010; 2252; 5156	9.96E-03	4.94E-02

Supplemental Table S3. Enriched gene ontology-based sets.

term_goid	term_ category	term_ level	term_name	members_input_overlap	members_input_overlap_geneids	p-value	q-value
GO:0042127	b	4	regulation of cell proliferation	590; 595; 652; 1000; 1602; 2119; 2192; 2252; 2254; 3552; 4254; 5156; 5176; 5732; 5742; 5745; 6387; 7010; 7022; 7049; 7168; 7291; 7472; 8654; 9037; 9510; 9536; 10252; 11095; 80310; 51176; 58189; 28514; 127435	PTH1R; PDGFRA; WFDC1; PTGS1; DACH1; SPRY1; BCHE; TPM1; IL1A; FBLN1; LEF1; PTGES; PODN; TEK; ADAMTS1; TWIST1; TFAP2C; ADAMTS8; PTGER2; CDH2; PDE5A; TGFBR3; PDGFD; DLL1; SERPINF1; FGF9; FGF7; KITLG; CXCL12; BMP4; ETV5; SEMA5A; CCND1; WNT2	1.12E-08	2.68E-06
GO:0051270	b	4	regulation of cellular component movement	652; 1602; 2192; 2252; 4254; 5156; 5176; 5337; 5919; 6387; 7010; 7049; 7168; 7291; 8434; 9037; 10154; 10507; 10512; 80310; 114757; 51176; 127435	RECK; PDGFRA; DACH1; FBLN1; SEMA4D; KITLG; PODN; TEK; TWIST1; TPM1; TGFBR3; PDGFD; CYGB; SERPINF1; FGF7; LEF1; CXCL12; PLXNC1; SEMA3C; BMP4; PLD1; SEMA5A; RARRES2	3.05E-08	2.68E-06
GO:2000145	b	4	regulation of cell motility	652; 1602; 2192; 2252; 4254; 5156; 5176; 5337; 5919; 6387; 7010; 7168; 7291; 8434; 9037; 10154; 10507; 10512; 80310; 114757; 51176; 127435	RECK; PDGFRA; DACH1; FBLN1; SEMA4D; KITLG; PODN; TEK; TWIST1; TPM1; PDGFD; CYGB; SERPINF1; FGF7; LEF1; CXCL12; PLXNC1; SEMA3C; BMP4; PLD1; SEMA5A; RARRES2	3.38E-08	2.68E-06
GO:0072001	b	4	renal system development	652; 445; 4093; 4811; 5156; 5176; 6943; 8516; 9510; 10252; 80310; 51176; 157506; 28514	ITGA8; PDGFRA; BMP4; ADAMTS1; SMAD9; RDH10; SPRY1; DLL1; NID1; SERPINF1; PDGFD; LEF1; ASS1; TCF21	3.55E-08	2.68E-06
GO:0001944	b	4	vasculature development	652; 1000; 2254; 3552; 5156; 5176; 6943; 7010; 7291; 7472; 8434; 9037; 10512; 144165; 80310; 51176; 51330; 23554; 58504; 28514	RECK; PDGFRA; BMP4; PDGFD; SEMA5A; PRICKLE1; TWIST1; WNT2; TSPAN12; SEMA3C; ARHGAP22; TEK; SERPINF1; FGF9; IL1A; LEF1; CDH2; TNFRSF12A; TCF21; DLL1	3.81E-08	2.68E-06
GO:0060485	b	4	mesenchyme development	652; 2254; 2295; 4254; 6943; 7049; 7291; 7472; 9037; 10507; 10512; 51176; 157506	TGFBR3; SEMA3C; BMP4; FGF9; SEMA5A; TWIST1; WNT2; RDH10; FOXF2; LEF1; KITLG; SEMA4D; TCF21	3.98E-08	2.68E-06
GO:0072358	b	4	cardiovascular system development	652; 1000; 2254; 3552; 5156; 5176; 6943; 7010; 7291; 7472; 8434; 9037; 10512; 144165; 80310; 51176; 51330; 23554; 58504; 28514	RECK; PDGFRA; BMP4; PDGFD; SEMA5A; PRICKLE1; TWIST1; WNT2; TSPAN12; SEMA3C; ARHGAP22; TEK; SERPINF1; FGF9; IL1A; LEF1; CDH2; TNFRSF12A; TCF21; DLL1	4.61E-08	2.68E-06
GO:0072359	b	4	circulatory system development	652; 1000; 2254; 3552; 5156; 5176; 6444; 6943; 7010; 7049; 7168; 7291; 7472; 8434; 9037; 9510; 10512; 144165; 80206; 80310; 51176; 51330; 23554; 58504; 28514	RECK; PDGFRA; TSPAN12; IL1A; TNFRSF12A; PRICKLE1; TEK; ADAMTS1; TWIST1; SGCD; TPM1; ARHGAP22; CDH2; TCF21; TGFBR3; PDGFD; DLL1; FHOD3; SERPINF1; FGF9; LEF1; SEMA3C; BMP4; SEMA5A; WNT2	4.61E-08	2.68E-06

Supplemental Table S3. Enriched gene ontology-based sets.

GO:0060537	b	4	muscle tissue development	652; 2254; 5156; 5307; 6444; 6943; 7049; 7168; 7291; 7472; 8516; 10512; 80206; 51176; 28514	TGFBR3; ITGA8; PDGFRA; BMP4; TWIST1; WNT2; SGCD; SEMA3C; TPM1; DLL1; FHOD3; FGF9; LEF1; PITX1; TCF21	9.63E-08	4.98E-06
GO:0001655	b	4	urogenital system development	652; 445; 4093; 4811; 5156; 5176; 6943; 8516; 9510; 10252; 80310; 51176; 157506; 28514	ITGA8; PDGFRA; BMP4; ADAMTS1; SMAD9; RDH10; SPRY1; DLL1; NID1; SERPINF1; PDGFD; LEF1; ASS1; TCF21	1.71E-07	7.97E-06
GO:0061458	b	4	reproductive system development	595; 652; 2252; 2254; 2295; 4254; 5156; 5176; 6943; 7022; 7472; 9510; 51176; 54477; 56977; 157506	PDGFRA; BMP4; ADAMTS1; FGF9; CCND1; WNT2; TFAP2C; PLEKHA5; SERPINF1; FOXF2; STOX2; FGF7; RDH10; KITLG; LEF1; TCF21	2.29E-07	9.70E-06
GO:0070848	b	4	response to growth factor	65997; 652; 2201; 2252; 2254; 2662; 3554; 4093; 5156; 7049; 7291; 7472; 8425; 8516; 10252; 80310; 51176; 56975; 28514	TGFBR3; ITGA8; PDGFRA; BMP4; SMAD9; RASL11B; TWIST1; WNT2; FAM20C; FBN2; DLL1; SPRY1; LTBP4; FGF9; FGF7; PDGFD; LEF1; IL1R1; GDF10	4.30E-07	1.67E-05
GO:0030154	b	4	cell differentiation	249; 590; 595; 652; 1000; 1066; 2114; 2119; 2192; 2201; 2254; 2273; 2295; 2662; 2947; 3552; 4147; 4254; 5156; 5176; 5307; 5745; 5919; 5997; 6387; 6444; 6943; 7010; 7049; 7168; 7225; 7291; 7472; 8425; 8516; 8654; 9037; 9510; 9770; 10154; 10507; 10512; 144165; 80206; 51176; 51330; 85464; 342035; 23682; 56975; 58504; 157506; 28514	PTH1R; ITGA8; PDGFRA; FAM20C; RASSF2; LTBP4; BCHE; IL1A; FBLN1; PITX1; SEMA4D; LEF1; PRICKLE1; TEK; ADAMTS1; GLDN; TWIST1; TNFRSF12A; SGCD; TPM1; RAB38; ETS2; ARHGAP22; FHL1; FOXF2; CDH2; TCF21; PDE5A; GDF10; TGFBR3; MATN2; RGS2; FBN2; CES1; DLL1; FHOD3; SERPINF1; FGF9; KITLG; CXCL12; PLXNC1; SEMA3C; BMP4; ETV5; SEMA5A; CCND1; WNT2; GSTM3; RARRES2; SSH2; RDH10; TRPC6; ALPL	5.75E-07	2.06E-05
GO:0051094	b	4	positive regulation of developmental process	595; 652; 1000; 2119; 2201; 2252; 2254; 3552; 4254; 5176; 5919; 6387; 7010; 7225; 7291; 7472; 8654; 9037; 9510; 10252; 10507; 80310; 51176; 51330; 56975; 28514	SPRY1; TRPC6; IL1A; SEMA4D; LEF1; TEK; ADAMTS1; TWIST1; TNFRSF12A; CDH2; PDE5A; PDGFD; FBN2; DLL1; SERPINF1; FGF9; FGF7; KITLG; CXCL12; BMP4; ETV5; SEMA5A; CCND1; WNT2; FAM20C; RARRES2	6.58E-07	2.19E-05
GO:0022603	b	4	regulation of anatomical structure morphogenesis	652; 1000; 2119; 2192; 2252; 3552; 5176; 6387; 7010; 7168; 7225; 7291; 7472; 9037; 10154; 10252; 10507; 10512; 144165; 51330; 85464; 23554; 28514	TSPAN12; SPRY1; TRPC6; IL1A; FBLN1; TNFRSF12A; PRICKLE1; TEK; TWIST1; SEMA4D; TPM1; CDH2; DLL1; SSH2; FGF7; CXCL12; PLXNC1; SEMA3C; BMP4; ETV5; SEMA5A; WNT2; SERPINF1	9.02E-07	2.80E-05
GO:0001501	b	4	skeletal system development	249; 652; 2114; 2201; 2254; 2662; 4093; 5156; 5307; 5745; 7010; 7291; 9770; 10507; 56975; 157506	PTH1R; PDGFRA; BMP4; SMAD9; TWIST1; RASSF2; FBN2; ETS2; TEK; RDH10; PITX1; FGF9; ALPL; SEMA4D; FAM20C; GDF10	1.12E-06	3.27E-05

Supplemental Table S3. Enriched gene ontology-based sets.

GO:0045595	b	4	regulation of cell differentiation	595; 652; 1000; 2119; 2192; 2201; 2254; 2662; 4254; 5156; 5176; 5919; 6387; 7225; 7291; 7472; 8425; 8654; 9037; 9510; 9770; 10154; 10507; 10512; 144165; 51176; 51330; 85464; 56975; 28514	PDGFRA; RASSF2; TRPC6; FBLN1; SEMA4D; LEF1; PRICKLE1; ADAMTS1; TWIST1; TNFRSF12A; CDH2; PDE5A; GDF10; LTBP4; FBN2; DLL1; SERPINF1; FGF9; KITLG; CXCL12; PLXNC1; SEMA3C; BMP4; ETV5; SEMA5A; CCND1; WNT2; FAM20C; RARRES2; SSH2	1.22E-06	3.34E-05
GO:0014031	b	4	mesenchymal cell development	4254; 7291; 9037; 10507; 10512; 51176; 157506	SEMA3C; SEMA5A; TWIST1; RDH10; LEF1; SEMA4D; KITLG	1.62E-06	4.18E-05
GO:0048864	b	4	stem cell development	4254; 7291; 9037; 10507; 10512; 51176; 157506	SEMA3C; SEMA5A; TWIST1; RDH10; LEF1; SEMA4D; KITLG	1.77E-06	4.34E-05
GO:0035108	b	4	limb morphogenesis	652; 2201; 2254; 5307; 7291; 8434; 10512; 51176; 157506	RECK; SEMA3C; BMP4; TWIST1; FBN2; RDH10; FGF9; LEF1; PITX1	2.18E-06	5.08E-05
GO:0060425	b	4	lung morphogenesis	652; 2252; 6943; 7472; 10252; 157506	BMP4; WNT2; SPRY1; RDH10; FGF7; TCF21	3.19E-06	7.07E-05
GO:0007517	b	4	muscle organ development	652; 445; 2254; 2273; 5307; 6444; 6943; 7049; 7168; 7291; 7472; 51176; 28514	TGFBR3; BMP4; TWIST1; WNT2; SGCD; TPM1; DLL1; FHL1; FGF9; LEF1; PITX1; ASS1; TCF21	3.98E-06	8.41E-05
GO:0072103	b	4	glomerulus vasculature morphogenesis	652; 5156; 6943	PDGFRA; BMP4; TCF21	4.42E-06	8.56E-05
GO:0072104	b	4	glomerular capillary formation	652; 5156; 6943	PDGFRA; BMP4; TCF21	4.42E-06	8.56E-05
GO:0048566	b	4	embryonic digestive tract development	2254; 2295; 5156; 5919; 6943	PDGFRA; RARRES2; FOXF2; FGF9; TCF21	5.03E-06	9.35E-05
GO:0048585	b	4	negative regulation of response to stimulus	65997; 595; 652; 1000; 2192; 2201; 2254; 3552; 5156; 5176; 5648; 5997; 6387; 6943; 7010; 7049; 7291; 8490; 9037; 9770; 10252; 10507; 10512; 144165; 51176; 58189; 127435	PDGFRA; RASSF2; SPRY1; IL1A; FBLN1; SEMA4D; PRICKLE1; TEK; TWIST1; RGS5; RGS2; CDH2; TCF21; TGFBR3; RASL11B; PODN; FBN2; SERPINF1; FGF9; WFDC1; LEF1; CXCL12; SEMA3C; BMP4; SEMA5A; CCND1; MASP1	5.52E-06	9.87E-05
GO:0035113	b	4	embryonic appendage morphogenesis	652; 2201; 2254; 5307; 7291; 8434; 51176; 157506	RECK; BMP4; TWIST1; FBN2; RDH10; FGF9; LEF1; PITX1	6.29E-06	1.06E-04
GO:0040017	b	4	positive regulation of locomotion	652; 2192; 2252; 4254; 5156; 5919; 6387; 7010; 7291; 9037; 10507; 10512; 80310; 51176	PDGFRA; BMP4; PDGFD; SEMA5A; TWIST1; RARRES2; SEMA3C; TEK; FGF7; LEF1; FBLN1; CXCL12; SEMA4D; KITLG	6.40E-06	1.06E-04
GO:0060173	b	4	limb development	652; 2201; 2254; 5307; 7291; 8434; 10512; 51176; 157506	RECK; SEMA3C; BMP4; TWIST1; FBN2; RDH10; FGF9; LEF1; PITX1	6.81E-06	1.07E-04
GO:0040013	b	4	negative regulation of locomotion	1602; 2192; 5176; 6387; 7168; 8434; 9037; 10507; 10512; 114757; 127435	RECK; SEMA3C; CYGB; SEMA5A; PODN; TPM1; SERPINF1; DACH1; FBLN1; CXCL12; SEMA4D	6.90E-06	1.07E-04

Supplemental Table S3. Enriched gene ontology-based sets.

GO:0061138	b	4	morphogenesis of a branching epithelium	652; 2119; 2252; 6943; 7472; 10252; 10512; 51176; 157506	SEMA3C; BMP4; ETV5; WNT2; SPRY1; RDH10; FGF7; LEF1; TCF21	9.44E-06	1.42E-04
GO:0008406	b	4	gonad development	595; 652; 2252; 2254; 4254; 5156; 6943; 7022; 9510; 157506	PDGFRA; BMP4; ADAMTS1; CCND1; TFAP2C; RDH10; FGF9; FGF7; KITLG; TCF21	9.78E-06	1.42E-04
GO:0051240	b	4	positive regulation of multicellular organismal process	595; 652; 2119; 2201; 2252; 2254; 3552; 4254; 5176; 5742; 5997; 6387; 7010; 7168; 7225; 7291; 7472; 8654; 9037; 9510; 10252; 10507; 80310; 51176; 51330; 56975; 28514	PTGS1; SPRY1; TRPC6; IL1A; SEMA4D; LEF1; TEK; ADAMTS1; TWIST1; TNFRSF12A; TPM1; RGS2; PDE5A; PDGFD; FBN2; DLL1; SERPINF1; FGF9; FGF7; KITLG; CXCL12; BMP4; ETV5; SEMA5A; CCND1; WNT2; FAM20C	1.06E-05	1.49E-04
GO:2000026	b	4	regulation of multicellular organismal development	595; 652; 1000; 2119; 2201; 2252; 2254; 3552; 4254; 5176; 6387; 7010; 7225; 7291; 7472; 9037; 9510; 9770; 10154; 10252; 10507; 10512; 144165; 80310; 51176; 51330; 85464; 23554; 56975; 28514	TSPAN12; RASSF2; SPRY1; TRPC6; IL1A; SEMA4D; LEF1; PRICKLE1; TEK; ADAMTS1; TWIST1; TNFRSF12A; CDH2; PDGFD; FBN2; DLL1; SERPINF1; FGF9; FGF7; KITLG; CXCL12; PLXNC1; SEMA3C; BMP4; ETV5; SEMA5A; CCND1; WNT2; FAM20C; SSH2	1.14E-05	1.57E-04
GO:0001654	b	4	eye development	652; 1415; 2201; 2254; 2295; 5156; 5176; 7291; 7472; 23554; 157506; 28514	PDGFRA; BMP4; FGF9; TWIST1; WNT2; TSPAN12; FBN2; CRYBB2; DLL1; SERPINF1; FOXF2; RDH10	1.19E-05	1.58E-04
GO:0009790	b	4	embryo development	652; 2114; 2201; 2254; 2295; 4254; 5156; 5307; 5919; 6943; 7168; 7291; 7472; 8434; 8516; 10512; 144165; 51176; 56977; 157506; 28514	ITGA8; RECK; PDGFRA; BMP4; FGF9; PRICKLE1; TWIST1; RARRES2; STOX2; FBN2; SEMA3C; TPM1; ETS2; DLL1; RDH10; FOXF2; WNT2; KITLG; PITX1; LEF1; TCF21	1.29E-05	1.66E-04
GO:0090287	b	4	regulation of cellular response to growth factor stimulus	65997; 652; 2201; 2254; 7049; 8425; 8516; 10252; 56975; 28514	TGFBR3; ITGA8; LTBP4; RASL11B; FAM20C; FBN2; DLL1; SPRY1; BMP4; FGF9	1.33E-05	1.67E-04
GO:0007167	b	4	enzyme linked receptor protein signaling pathway	65997; 652; 2051; 2201; 2252; 2254; 2662; 2674; 4093; 5156; 7010; 7049; 8425; 8516; 9770; 10252; 80310; 51176; 55824; 56975; 28514	TGFBR3; ITGA8; PDGFRA; BMP4; EPHB6; RASL11B; PAG1; RASSF2; FBN2; LTBP4; DLL1; SPRY1; TEK; GFRA1; SMAD9; FGF9; FGF7; PDGFD; LEF1; FAM20C; GDF10	1.39E-05	1.70E-04

Supplemental Table S3. Enriched gene ontology-based sets.

GO:0048523	b	4	negative regulation of cellular process	65997; 590; 595; 652; 1000; 445; 1602; 2114; 2192; 2201; 2254; 2273; 2295; 2662; 3552; 4254; 4314; 4774; 5156; 5176; 5307; 5648; 5742; 5745; 5997; 6387; 6943; 7010; 7022; 7025; 7049; 7168; 7225; 7291; 8434; 8490; 8654; 8989; 9037; 9510; 9536; 9770; 10252; 10507; 10512; 11095; 144165; 80206; 114757; 51176; 58189; 55824; 28514; 127435; 30061	PTH1R; RECK; PDGFRA; RGS2; PAG1; RASSF2; PTGS1; WFDC1; SPRY1; BCHE; TRPA1; IL1A; FBLN1; PITX1; ASS1; PTGES; PRICKLE1; TEK; ADAMTS1; TWIST1; SEMA4D; TFAP2C; NR2F1; ADAMTS8; TPM1; ETS2; RGS5; FHL1; FOXF2; LEF1; CDH2; TCF21; PDE5A; GDF10; TGFBR3; RASL11B; PODN; FBN2; SEMA3C; DLL1; FHOD3; SERPINF1; CYGB; FGF9; DACH1; KITLG; CXCL12; NFIA; BMP4; SLC40A1; SEMA5A; CCND1; MASP1; TRPC6; MMP3	1.97E-05	2.35E-04
GO:0010646	b	4	regulation of cell communication	65997; 590; 595; 652; 1000; 1075; 2040; 2192; 2201; 2252; 2254; 2662; 3552; 4254; 5156; 5742; 5997; 6387; 6574; 6943; 7010; 7049; 7291; 7472; 8425; 8490; 8516; 8654; 9037; 9770; 10252; 10507; 144165; 80310; 51176; 51330; 22829; 55824; 56975; 58504; 9627; 28514; 127435	ITGA8; PDGFRA; PAG1; RASSF2; PTGS1; STOM; SPRY1; BCHE; IL1A; FBLN1; SEMA4D; LEF1; PRICKLE1; TEK; SNCAIP; ARHGAP22; TWIST1; TNFRSF12A; RGS5; RGS2; CDH2; TCF21; PDE5A; GDF10; TGFBR3; LTBP4; PDGFD; RASL11B; PODN; NLGN4Y; CTSC; FBN2; DLL1; SLC20A1; FGF9; FGF7; KITLG; CXCL12; BMP4; SEMA5A; CCND1; WNT2; FAM20C	2.16E-05	2.51E-04
GO:0048017	b	4	inositol lipid-mediated signaling	2252; 2254; 4254; 5156; 5337; 7010; 7291; 10507; 80310	PDGFRA; TEK; PDGFD; PLD1; TWIST1; FGF9; FGF7; KITLG; SEMA4D	2.60E-05	2.95E-04
GO:0072012	b	4	glomerulus vasculature development	652; 5156; 6943; 80310	PDGFRA; BMP4; PDGFD; TCF21	2.66E-05	2.95E-04
GO:0060541	b	4	respiratory system development	652; 445; 2252; 2254; 6943; 7472; 10252; 51176; 157506	BMP4; WNT2; SPRY1; RDH10; FGF9; FGF7; LEF1; ASS1; TCF21	3.16E-05	3.41E-04
GO:0001667	b	4	ameboidal-type cell migration	652; 2252; 4254; 5176; 7010; 7291; 9037; 10507; 10512; 114757; 51176	SEMA3C; TEK; SEMA5A; TWIST1; BMP4; SERPINF1; CYGB; FGF7; KITLG; SEMA4D; LEF1	3.84E-05	4.05E-04
GO:0071560	b	4	cellular response to transforming growth factor beta stimulus	65997; 2201; 2662; 4093; 7049; 7472; 8425; 8516; 80310	TGFBR3; ITGA8; LTBP4; PDGFD; RASL11B; WNT2; FBN2; GDF10; SMAD9	4.10E-05	4.23E-04
GO:0002009	b	4	morphogenesis of an epithelium	652; 2119; 2252; 2295; 6943; 7291; 7472; 10252; 10512; 144165; 51176; 157506; 28514; 94234	FOXQ1; PRICKLE1; SEMA3C; BMP4; ETV5; TWIST1; WNT2; DLL1; SPRY1; RDH10; FOXF2; FGF7; LEF1; TCF21	4.59E-05	4.64E-04
GO:0009966	b	4	regulation of signal transduction	65997; 595; 652; 1000; 1075; 2040; 2192; 2201; 2252; 2254; 2662; 3552; 4254; 5156; 5997; 6387; 6574; 6943; 7010; 7049; 7291; 7472; 8425; 8490; 8516; 8654; 9037; 9770; 10252; 10507; 144165; 80310; 51176; 51330; 55824; 56975; 58504; 28514; 127435	ITGA8; PDGFRA; PAG1; RASSF2; STOM; SPRY1; IL1A; FBLN1; SEMA4D; LEF1; PRICKLE1; TEK; ARHGAP22; TWIST1; TNFRSF12A; RGS5; RGS2; CDH2; TCF21; PDE5A; GDF10; TGFBR3; LTBP4; PDGFD; RASL11B; PODN; CTSC; FBN2; DLL1; SLC20A1; FGF9; FGF7; KITLG; CXCL12; BMP4; SEMA5A; CCND1; WNT2; FAM20C	5.20E-05	5.14E-04

Supplemental Table S3. Enriched gene ontology-based sets.

GO:0007162	b	4	negative regulation of cell adhesion	652; 445; 2192; 6387; 8654; 9037; 10507; 51176; 55824	BMP4; SEMA5A; SEMA4D; PAG1; FBLN1; CXCL12; ASS1; LEF1; PDE5A	5.45E-05	5.18E-04
GO:0003007	b	4	heart morphogenesis	652; 7010; 7049; 7168; 7291; 7472; 9510; 10512; 28514	TGFBR3; SEMA3C; TEK; ADAMTS1; TWIST1; WNT2; TPM1; DLL1; BMP4	5.45E-05	5.18E-04
GO:0001656	b	4	metanephros development	652; 5156; 6943; 8516; 10252; 157506	ITGA8; PDGFRA; BMP4; SPRY1; RDH10; TCF21	6.05E-05	5.62E-04
GO:0060429	b	4	epithelium development	595; 652; 1000; 1066; 2119; 2252; 2295; 2947; 4093; 6943; 7291; 7472; 10252; 10512; 144165; 51176; 56975; 157506; 28514; 94234; 30061	FAM20C; PRICKLE1; SEMA3C; BMP4; ETV5; SMAD9; DLL1; CCND1; WNT2; GSTM3; CES1; TWIST1; SPRY1; FOXQ1; RDH10; FOXF2; FGF7; LEF1; CDH2; TCF21; SLC40A1	6.66E-05	6.07E-04
GO:0060502	b	4	epithelial cell proliferation involved in lung morphogenesis	652; 2252; 7472	FGF7; WNT2; BMP4	7.04E-05	6.30E-04
GO:0048562	b	4	embryonic organ morphogenesis	652; 2201; 2254; 2295; 5156; 6943; 7291; 8516; 157506; 28514	ITGA8; PDGFRA; BMP4; FGF9; TWIST1; FBN2; DLL1; RDH10; FOXF2; TCF21	7.34E-05	6.44E-04
GO:0071310	b	4	cellular response to organic substance	249; 65997; 595; 652; 445; 2201; 2252; 2254; 2662; 3552; 3554; 4093; 5156; 5176; 5732; 5919; 6387; 6943; 7025; 7049; 7291; 7472; 8425; 8516; 9510; 10252; 80310; 51176; 51330; 56975; 81035; 28514; 127435	ITGA8; PDGFRA; SMAD9; SPRY1; IL1A; ASS1; IL1R1; PODN; ADAMTS1; TWIST1; TNFRSF12A; NR2F1; PTGER2; TCF21; GDF10; TGFBR3; LTBP4; PDGFD; RASL11B; FBN2; DLL1; SERPINF1; FGF9; FGF7; LEF1; CXCL12; BMP4; CCND1; WNT2; FAM20C; RARRES2; COLEC12; ALPL	9.08E-05	7.82E-04
GO:1901654	b	4	response to ketone	595; 652; 445; 5176; 5732; 5742; 7049; 9510	TGFBR3; PTGS1; BMP4; ADAMTS1; CCND1; PTGER2; SERPINF1; ASS1	9.42E-05	7.82E-04
GO:0050920	b	4	regulation of chemotaxis	5156; 5919; 6387; 9037; 10154; 10507; 10512; 80310	PLXNC1; PDGFRA; PDGFD; SEMA5A; RARRES2; SEMA3C; CXCL12; SEMA4D	9.42E-05	7.82E-04
GO:0048638	b	4	regulation of developmental growth	652; 2254; 6387; 7472; 9037; 10154; 10507; 10512; 51330; 28514	PLXNC1; SEMA3C; BMP4; SEMA5A; WNT2; DLL1; TNFRSF12A; FGF9; CXCL12; SEMA4D	1.00E-04	8.19E-04
GO:0060562	b	4	epithelial tube morphogenesis	652; 2119; 6943; 7291; 7472; 10252; 144165; 51176; 157506; 28514	PRICKLE1; BMP4; ETV5; TWIST1; WNT2; DLL1; SPRY1; RDH10; LEF1; TCF21	1.28E-04	1.03E-03
GO:0048754	b	4	branching morphogenesis of an epithelial tube	652; 2119; 6943; 7472; 10252; 51176; 157506	BMP4; ETV5; WNT2; SPRY1; RDH10; LEF1; TCF21	1.32E-04	1.04E-03
GO:0090596	b	4	sensory organ morphogenesis	652; 2201; 2254; 2295; 7291; 7472; 8516; 23554; 28514	ITGA8; BMP4; FGF9; TWIST1; WNT2; TSPAN12; FBN2; DLL1; FOXF2	1.40E-04	1.08E-03
GO:0060347	b	4	heart trabecula formation	7010; 7049; 9510	TGFBR3; TEK; ADAMTS1	1.53E-04	1.16E-03

Supplemental Table S3. Enriched gene ontology-based sets.

GO:1901861	b	4	regulation of muscle tissue development	652; 2254; 7291; 7472; 51176; 28514	BMP4; TWIST1; WNT2; DLL1; FGF9; LEF1	1.61E-04	1.20E-03
GO:0055123	b	4	digestive system development	652; 445; 2254; 2295; 5156; 5919; 6943	PDGFRA; BMP4; FGF9; RARRES2; FOXF2; ASS1; TCF21	1.63E-04	1.20E-03
GO:0072275	b	4	metanephric glomerulus morphogenesis	5156; 6943	PDGFRA; TCF21	1.76E-04	1.26E-03
GO:0030198	b	4	extracellular matrix organization	1287; 2192; 2201; 2295; 4314; 4811; 8434; 8516; 11096; 169611	ITGA8; RECK; OLFML2A; FBN2; NID1; COL4A5; FOXF2; ADAMTS5; FBLN1; MMP3	1.76E-04	1.26E-03
GO:0071526	b	4	semaphorin-plexin signaling pathway	9037; 10154; 10507; 10512	PLXNC1; SEMA3C; SEMA4D; SEMA5A	1.83E-04	1.29E-03
GO:0002040	b	4	sprouting angiogenesis	652; 7010; 9037; 51176; 28514	TEK; LEF1; BMP4; SEMA5A; DLL1	2.04E-04	1.42E-03
GO:0046545	b	4	development of primary female sexual characteristics	652; 1602; 2252; 4254; 5156; 9510	PDGFRA; BMP4; ADAMTS1; FGF7; DACH1; KITLG	2.41E-04	1.65E-03
GO:0048545	b	4	response to steroid hormone	249; 590; 595; 652; 445; 5176; 5732; 5742; 6943; 7025; 51176	PTGS1; BMP4; CCND1; NR2F1; PTGER2; BCHE; SERPINF1; LEF1; ASS1; TCF21; ALPL	2.57E-04	1.73E-03
GO:0042476	b	4	odontogenesis	249; 652; 7291; 9510; 51176; 56975	BMP4; ADAMTS1; TWIST1; FAM20C; LEF1; ALPL	2.65E-04	1.76E-03
GO:0000302	b	4	response to reactive oxygen species	652; 3554; 4314; 5156; 7168; 7225; 8989; 80310	PDGFRA; BMP4; PDGFD; TPM1; TRPC6; TRPA1; MMP3; IL1R1	2.99E-04	1.96E-03
GO:0014070	b	4	response to organic cyclic compound	249; 590; 595; 652; 445; 4093; 5176; 5732; 5742; 6943; 7010; 7025; 8989; 9510; 9536; 51176; 58189; 81035	COLEC12; PTGS1; BMP4; ADAMTS1; SMAD9; CCND1; NR2F1; PTGER2; LEF1; TEK; BCHE; TRPA1; WFDC1; SERPINF1; PTGES; ASS1; TCF21; ALPL	3.68E-04	2.38E-03
GO:0023057	b	4	negative regulation of signaling	65997; 590; 595; 652; 1000; 2192; 2201; 2254; 3552; 5742; 5997; 6387; 6943; 7049; 7291; 8490; 9770; 10252; 144165; 51176; 127435	TGFBR3; PRICKLE1; PTGS1; BMP4; RASL11B; PODN; RGS2; CCND1; RASSF2; FBN2; SPRY1; TWIST1; CXCL12; BCHE; FGF9; IL1A; FBLN1; CDH2; LEF1; TCF21; RGS5	3.77E-04	2.40E-03
GO:0015669	b	4	gas transport	3047; 3048; 114757	CYGB; HBG2; HBG1	3.95E-04	2.48E-03
GO:0007399	b	4	nervous system development	590; 652; 1000; 2119; 2254; 2674; 2947; 4093; 4147; 4774; 5176; 5307; 6387; 7225; 7291; 7472; 8516; 9037; 9510; 10154; 10507; 10512; 144165; 51176; 51330; 85464; 342035; 54510; 29116; 22829; 28514	ITGA8; SMAD9; MYLIP; TRPC6; PITX1; SEMA4D; PRICKLE1; ADAMTS1; GLDN; TWIST1; TNFRSF12A; CDH2; MATN2; NLGN4Y; SEMA3C; DLL1; SERPINF1; FGF9; LEF1; CXCL12; PLXNC1; NFIA; BMP4; ETV5; SEMA5A; WNT2; GSTM3; SSH2; GFRA1; BCHE; PCDH18	4.02E-04	2.49E-03
GO:0046660	b	4	female sex differentiation	652; 1602; 2252; 4254; 5156; 9510	PDGFRA; BMP4; ADAMTS1; FGF7; DACH1; KITLG	4.51E-04	2.76E-03

Supplemental Table S3. Enriched gene ontology-based sets.

GO:0031100	b	4	animal organ regeneration	595; 6387; 7010; 7049; 51176	CCND1; TEK; LEF1; CXCL12; TGFBR3	4.78E-04	2.88E-03
GO:0030278	b	4	regulation of ossification	652; 2201; 2662; 7291; 9770; 10507; 56975	BMP4; TWIST1; RASSF2; FBN2; FAM20C; SEMA4D; GDF10	4.94E-04	2.94E-03
GO:0070542	b	4	response to fatty acid	445; 5732; 5742; 7049; 9510	TGFBR3; ADAMTS1; PTGS1; ASS1; PTGER2	5.04E-04	2.97E-03
GO:0071731	b	4	response to nitric oxide	652; 3554; 4314	BMP4; MMP3; IL1R1	5.37E-04	3.12E-03
GO:0045927	b	4	positive regulation of growth	2254; 6387; 7472; 9037; 10507; 51176; 51330; 28514	SEMA5A; WNT2; DLL1; TNFRSF12A; FGF9; LEF1; CXCL12; SEMA4D	5.50E-04	3.14E-03
GO:0031214	b	4	biomineral tissue development	249; 652; 2201; 5745; 7291; 56975	PTH1R; BMP4; TWIST1; FAM20C; FBN2; ALPL	5.54E-04	3.14E-03
GO:0006931	b	4	substrate-dependent cell migration, cell attachment to substrate	2192; 51330	FBLN1; TNFRSF12A	5.79E-04	3.21E-03
GO:0060431	b	4	primary lung bud formation	7472; 157506	WNT2; RDH10	5.79E-04	3.21E-03
GO:0051241	b	4	negative regulation of multicellular organismal process	652; 2192; 2662; 5156; 5176; 5997; 7010; 7225; 7291; 8654; 9037; 10507; 10512; 144165; 114757; 51176; 29116; 28514	PRICKLE1; PDGFRA; BMP4; SEMA5A; TWIST1; SEMA3C; MYLIP; DLL1; TEK; SERPINF1; CYGB; RGS2; TRPC6; FBLN1; SEMA4D; LEF1; PDE5A; GDF10	6.74E-04	3.69E-03
GO:0060688	b	4	regulation of morphogenesis of a branching structure	652; 2119; 2252; 7472	FGF7; WNT2; BMP4; ETV5	7.36E-04	3.93E-03
GO:0050918	b	4	positive chemotaxis	652; 2252; 6387; 9037	FGF7; CXCL12; BMP4; SEMA5A	7.36E-04	3.93E-03
GO:0048522	b	4	positive regulation of cellular process	595; 652; 1000; 1075; 445; 2040; 2051; 2114; 2119; 2192; 2201; 2252; 2254; 2295; 2662; 3552; 4093; 4254; 4314; 4774; 5156; 5176; 5307; 5745; 5919; 5997; 6387; 6574; 6943; 7010; 7022; 7049; 7168; 7225; 7291; 7472; 8516; 8654; 9037; 9510; 9770; 10507; 10512; 144165; 80310; 51176; 51330; 55824; 56975; 387496; 81035; 28514; 30061	PTH1R; ITGA8; PDGFRA; SMAD9; PAG1; RASSF2; STOM; TRPC6; IL1A; FBLN1; PITX1; ASS1; LEF1; PRICKLE1; TEK; ADAMTS1; TWIST1; SEMA4D; TFAP2C; TPM1; ETS2; FOXF2; CDH2; TCF21; PDE5A; GDF10; TGFBR3; EPHB6; RGS2; RASL11A; CTSC; FBN2; SEMA3C; DLL1; SLC20A1; SERPINF1; FGF9; FGF7; KITLG; CXCL12; NFIA; BMP4; ETV5; SLC40A1; SEMA5A; CCND1; WNT2; FAM20C; RARRES2; TNFRSF12A; COLEC12; PDGFD; MMP3	7.59E-04	4.01E-03
GO:0001558	b	4	regulation of cell growth	2273; 6387; 8425; 9037; 10154; 10507; 10512; 51176; 51330; 58189	PLXNC1; SEMA3C; LTBP4; SEMA5A; TNFRSF12A; FHL1; WFDC1; LEF1; CXCL12; SEMA4D	9.83E-04	5.10E-03

Supplemental Table S3. Enriched gene ontology-based sets.

GO:0000165	b	4	MAPK cascade	652; 1000; 2192; 2252; 2254; 2662; 2674; 3552; 4254; 5156; 5997; 7010; 8654; 9770; 10252; 80310	PDGFRA; BMP4; PDGFD; RGS2; RASSF2; SPRY1; TEK; GFRA1; FGF9; FGF7; IL1A; FBLN1; CDH2; KITLG; PDE5A; GDF10	9.96E-04	5.10E-03
GO:0042060	b	4	wound healing	652; 2192; 2252; 3047; 3048; 3552; 5156; 7168; 7225; 80310; 51330; 58189	PDGFRA; BMP4; PDGFD; HBG2; HBG1; WFDC1; TRPC6; TPM1; FGF7; IL1A; FBLN1; TNFRSF12A	1.00E-03	5.10E-03
GO:0036075	b	4	replacement ossification	249; 652; 7010	TEK; BMP4; ALPL	1.02E-03	5.10E-03
GO:0061384	b	4	heart trabecula morphogenesis	7010; 7049; 9510	TGFBR3; TEK; ADAMTS1	1.02E-03	5.10E-03
GO:1990138	b	4	neuron projection extension	6387; 9037; 10154; 10507; 10512; 51330	PLXNC1; SEMA3C; SEMA5A; SEMA4D; CXCL12; TNFRSF12A	1.12E-03	5.53E-03
GO:0055001	b	4	muscle cell development	652; 5156; 6444; 7168; 80206; 51176	PDGFRA; FHOD3; SGCD; TPM1; BMP4; LEF1	1.16E-03	5.66E-03
GO:0048705	b	4	skeletal system morphogenesis	249; 652; 2201; 5156; 7010; 7291; 157506	PDGFRA; TEK; TWIST1; FBN2; BMP4; RDH10; ALPL	1.19E-03	5.75E-03
GO:0032101	b	4	regulation of response to external stimulus	2040; 2252; 3554; 5156; 5176; 5648; 5919; 6387; 7010; 9037; 10154; 10507; 10512; 80310; 58189	PLXNC1; PDGFRA; TEK; PDGFD; SEMA5A; RARRES2; MASP1; STOM; SERPINF1; WFDC1; FGF7; CXCL12; SEMA4D; SEMA3C; IL1R1	1.47E-03	7.04E-03
GO:0008585	b	4	female gonad development	652; 2252; 4254; 5156; 9510	FGF7; KITLG; BMP4; ADAMTS1; PDGFRA	1.49E-03	7.04E-03
GO:0051128	b	4	regulation of cellular component organization	652; 1000; 2040; 2119; 2192; 2273; 3552; 4314; 5156; 5176; 5337; 5997; 6387; 7010; 7168; 7225; 7291; 8425; 9037; 9510; 10154; 10507; 10512; 80206; 51176; 51330; 85464; 58189; 9627; 28514	PDGFRA; STOM; TRPC6; IL1A; FBLN1; SEMA4D; TEK; ADAMTS1; SNCAIP; TWIST1; TNFRSF12A; TPM1; FHL1; RGS2; CDH2; LTBP4; DLL1; FHOD3; SERPINF1; WFDC1; LEF1; CXCL12; PLXNC1; SEMA3C; BMP4; ETV5; PLD1; SEMA5A; SSH2; MMP3	1.50E-03	7.04E-03
GO:0034599	b	4	cellular response to oxidative stress	652; 2119; 4314; 5156; 7168; 7225; 80310	PDGFRA; BMP4; ETV5; TPM1; TRPC6; PDGFD; MMP3	1.68E-03	7.81E-03
GO:0009893	b	4	positive regulation of metabolic process	595; 652; 1000; 1075; 445; 2114; 2119; 2192; 2252; 2254; 2295; 2662; 3552; 4093; 4254; 4774; 5156; 5307; 5745; 5919; 6943; 7010; 7022; 7225; 7291; 7472; 8516; 8654; 9770; 10507; 144165; 80310; 51176; 29116; 387496; 28514; 30061	PTH1R; ITGA8; PDGFRA; SMAD9; RASSF2; TRPC6; IL1A; FBLN1; PITX1; ASS1; LEF1; PRICKLE1; TEK; TWIST1; SEMA4D; TFAP2C; ETS2; FOXF2; CDH2; TCF21; PDE5A; GDF10; PDGFD; RASL11A; CTSC; DLL1; MYLIP; FGF9; FGF7; KITLG; NFIA; BMP4; ETV5; SLC40A1; CCND1; WNT2; RARRES2	1.74E-03	8.01E-03
GO:0003401	b	4	axis elongation	652; 10252; 157506	SPRY1; BMP4; RDH10	1.88E-03	8.57E-03
GO:0033993	b	4	response to lipid	249; 590; 595; 652; 445; 5176; 5732; 5742; 6943; 7025; 7049; 7472; 9510; 9536; 51176; 58189	TGFBR3; PTGS1; BMP4; ADAMTS1; CCND1; WNT2; NR2F1; PTGER2; LEF1; BCHE; WFDC1; SERPINF1; PTGES; ASS1; TCF21; ALPL	1.97E-03	8.88E-03

Supplemental Table S3. Enriched gene ontology-based sets.

GO:0044259	b	4	multicellular organismal macromolecule metabolic process	652; 1287; 4314; 114757; 64066	MMP27; CYGB; COL4A5; BMP4; MMP3	2.04E-03	9.05E-03
GO:0060363	b	4	cranial suture morphogenesis	652; 7291	TWIST1; BMP4	2.04E-03	9.05E-03
GO:0001935	b	4	endothelial cell proliferation	652; 6387; 7010; 7472; 9037	TEK; CXCL12; BMP4; SEMA5A; WNT2	2.12E-03	9.29E-03
GO:0008361	b	4	regulation of cell size	6387; 9037; 10154; 10507; 10512; 51330	PLXNC1; SEMA3C; SEMA5A; SEMA4D; CXCL12; TNFRSF12A	2.37E-03	1.03E-02
GO:0034694	b	4	response to prostaglandin	5732; 7049; 9510	TGFBR3; ADAMTS1; PTGER2	2.44E-03	1.05E-02
GO:0072110	b	4	glomerular mesangial cell proliferation	652; 80310	BMP4; PDGFD	2.54E-03	1.08E-02
GO:0031076	b	4	embryonic camera-type eye development	2295; 7291; 157506	TWIST1; RDH10; FOXF2	2.65E-03	1.12E-02
GO:0071229	b	4	cellular response to acid chemical	445; 5176; 5732; 7472; 9510; 80310	ADAMTS1; WNT2; PTGER2; SERPINF1; PDGFD; ASS1	3.04E-03	1.27E-02
GO:0016337	b	4	single organismal cell-cell adhesion	652; 1000; 445; 2051; 5156; 6387; 8516; 8654; 10507; 51176; 342035; 22829; 55824	ITGA8; PDGFRA; CDH2; EPHB6; GLDN; SEMA4D; NLGN4Y; BMP4; PAG1; LEF1; CXCL12; ASS1; PDE5A	3.23E-03	1.34E-02
GO:0000902	b	4	cell morphogenesis	1000; 2192; 4147; 6387; 7010; 7168; 7225; 8516; 9037; 10154; 10507; 10512; 51176; 51330; 85464	ITGA8; PLXNC1; SEMA3C; TEK; SEMA5A; TNFRSF12A; TPM1; CXCL12; MATN2; TRPC6; SSH2; FBLN1; CDH2; SEMA4D; LEF1	3.65E-03	1.47E-02
GO:0008584	b	4	male gonad development	595; 2254; 4254; 6943; 7022	CCND1; KITLG; TCF21; FGF9; TFAP2C	3.68E-03	1.47E-02
GO:0046546	b	4	development of primary male sexual characteristics	595; 2254; 4254; 6943; 7022	CCND1; KITLG; TCF21; FGF9; TFAP2C	3.68E-03	1.47E-02
GO:0006069	b	4	ethanol oxidation	124; 125	ADH1B; ADH1A	3.69E-03	1.47E-02
GO:0040034	b	4	regulation of development, heterochronic	2254; 28514	DLL1; FGF9	3.69E-03	1.47E-02
GO:0072210	b	4	metanephric nephron development	652; 5156; 6943	PDGFRA; BMP4; TCF21	3.84E-03	1.50E-02
GO:0050919	b	4	negative chemotaxis	9037; 10507; 10512	SEMA3C; SEMA4D; SEMA5A	3.84E-03	1.50E-02
GO:0060349	b	4	bone morphogenesis	249; 652; 7010; 7291	TWIST1; TEK; BMP4; ALPL	3.87E-03	1.50E-02
GO:0030879	b	4	mammary gland development	595; 652; 2119; 7472; 51176	CCND1; WNT2; LEF1; BMP4; ETV5	3.93E-03	1.51E-02

Supplemental Table S3. Enriched gene ontology-based sets.

GO:0051093	b	4	negative regulation of developmental process	595; 652; 2192; 2662; 5176; 7010; 7225; 7291; 9037; 10507; 10512; 144165; 51176; 28514	PRICKLE1; SEMA3C; TEK; SEMA5A; DLL1; CCND1; TWIST1; BMP4; SERPINF1; TRPC6; FBLN1; SEMA4D; LEF1; GDF10	4.33E-03	1.64E-02
GO:0061430	b	4	bone trabecula morphogenesis	2201; 10507	SEMA4D; FBN2	4.34E-03	1.64E-02
GO:0043583	b	4	ear development	652; 2254; 7291; 8516; 157506; 28514	ITGA8; BMP4; TWIST1; DLL1; RDH10; FGF9	4.79E-03	1.79E-02
GO:0048584	b	4	positive regulation of response to stimulus	652; 1000; 1075; 2254; 2662; 3552; 4254; 4314; 5156; 5648; 5919; 6387; 6574; 7010; 7049; 7472; 8516; 8654; 9037; 9770; 10507; 80310; 51330; 55824; 81035; 28514	ITGA8; PDGFRA; PAG1; RASSF2; IL1A; SEMA4D; TEK; TNFRSF12A; CDH2; PDE5A; GDF10; TGFBR3; PDGFD; CTSC; DLL1; SLC20A1; FGF9; KITLG; CXCL12; BMP4; SEMA5A; WNT2; RARRES2; MASP1; COLEC12; MMP3	4.90E-03	1.82E-02
GO:0016331	b	4	morphogenesis of embryonic epithelium	652; 7291; 7472; 144165; 157506	TWIST1; WNT2; BMP4; RDH10; PRICKLE1	5.31E-03	1.96E-02
GO:0022617	b	4	extracellular matrix disassembly	2201; 4314; 4811; 11096	ADAMTS5; NID1; FBN2; MMP3	5.36E-03	1.96E-02
GO:0010623	b	4	programmed cell death involved in cell development	3552; 4254	IL1A; KITLG	5.78E-03	2.10E-02
GO:0071773	b	4	cellular response to BMP stimulus	652; 2662; 4093; 7049; 51176	TGFBR3; LEF1; BMP4; SMAD9; GDF10	6.11E-03	2.20E-02
GO:0048546	b	4	digestive tract morphogenesis	652; 5156; 6943	PDGFRA; BMP4; TCF21	6.35E-03	2.27E-02
GO:0072224	b	4	metanephric glomerulus development	5156; 6943	PDGFRA; TCF21	6.58E-03	2.33E-02
GO:0023056	b	4	positive regulation of signaling	652; 1000; 1075; 2254; 2662; 3552; 4254; 5156; 6574; 7010; 7049; 7472; 8516; 8654; 9037; 9770; 10507; 80310; 51330; 55824; 28514	TGFBR3; ITGA8; PDGFRA; BMP4; RASSF2; SEMA5A; WNT2; PDGFD; CTSC; TEK; DLL1; TNFRSF12A; SLC20A1; FGF9; PAG1; IL1A; KITLG; CDH2; SEMA4D; PDE5A; GDF10	6.61E-03	2.33E-02
GO:0072164	b	4	mesonephric tubule development	652; 4093; 6943; 10252	SPRY1; BMP4; SMAD9; TCF21	6.96E-03	2.43E-02
GO:0046661	b	4	male sex differentiation	595; 2254; 4254; 6943; 7022	CCND1; KITLG; TCF21; FGF9; TFAP2C	7.00E-03	2.43E-02
GO:0048872	b	4	homeostasis of number of cells	652; 1000; 4254; 7049; 9770; 30061	TGFBR3; BMP4; SLC40A1; RASSF2; KITLG; CDH2	7.34E-03	2.53E-02
GO:0014855	b	4	striated muscle cell proliferation	2254; 7049; 7472	TGFBR3; WNT2; FGF9	7.49E-03	2.56E-02
GO:0071466	b	4	cellular response to xenobiotic stimulus	1066; 2947; 5742; 119391	CES1; GSTO2; PTGS1; GSTM3	7.72E-03	2.62E-02

Supplemental Table S3. Enriched gene ontology-based sets.

GO:0097485	b	4	neuron projection guidance	4147; 6387; 9037; 10154; 10507; 10512	PLXNC1; SEMA3C; MATN2; SEMA5A; CXCL12; SEMA4D	7.82E-03	2.64E-02
GO:0001823	b	4	mesonephros development	652; 4093; 6943; 10252	SPRY1; BMP4; SMAD9; TCF21	7.99E-03	2.67E-02
GO:0030239	b	4	myofibril assembly	5156; 7168; 80206	PDGFRA; FHOD3; TPM1	8.32E-03	2.76E-02
GO:0055006	b	4	cardiac cell development	652; 5156; 80206	FHOD3; PDGFRA; BMP4	9.19E-03	3.03E-02
GO:0010632	b	4	regulation of epithelial cell migration	652; 2252; 5176; 7010; 9037	FGF7; TEK; BMP4; SERPINF1; SEMA5A	9.50E-03	3.11E-02
GO:0060326	b	4	cell chemotaxis	5156; 5919; 6387; 9037; 80310; 51176	PDGFRA; PDGFD; SEMA5A; RARRES2; LEF1; CXCL12	9.97E-03	3.24E-02
